# Mechanisms of ischaemia-induced arrhythmias in hypertrophic cardiomyopathy: a large-scale computational study

**DOI:** 10.1093/cvr/cvae086

**Published:** 2024-04-22

**Authors:** James A Coleman, Ruben Doste, Zakariye Ashkir, Raffaele Coppini, Rafael Sachetto, Hugh Watkins, Betty Raman, Alfonso Bueno-Orovio

**Affiliations:** Department of Computer Science, University of Oxford, Oxford, UK; Department of Computer Science, University of Oxford, Oxford, UK; Oxford Centre for Clinical Magnetic Resonance Research (OCMR), Radcliffe Department of Medicine, Division of Cardiovascular Medicine, University of Oxford, Oxford, UK; Department of NeuroFarBa, University of Florence, Florence, Italy; Department of Computer Science, Federal University of São João del-Rei, São João del-Rei, Minas Gerais, Brazil; Radcliffe Department of Medicine, Division of Cardiovascular Medicine, University of Oxford, Oxford, UK; Oxford Centre for Clinical Magnetic Resonance Research (OCMR), Radcliffe Department of Medicine, Division of Cardiovascular Medicine, University of Oxford, Oxford, UK; Department of Computer Science, University of Oxford, Oxford, UK

**Keywords:** Hypertrophic cardiomyopathy, Ischaemia, Arrhythmic risk, Modelling and simulation

## Abstract

**Aims:**

Lethal arrhythmias in hypertrophic cardiomyopathy (HCM) are widely attributed to myocardial ischaemia and fibrosis. How these factors modulate arrhythmic risk remains largely unknown, especially as invasive mapping protocols are not routinely used in these patients. By leveraging multiscale digital twin technologies, we aim to investigate ischaemic mechanisms of increased arrhythmic risk in HCM.

**Methods and results:**

Computational models of human HCM cardiomyocytes, tissue, and ventricles were used to simulate outcomes of Phase 1A acute myocardial ischaemia. Cellular response predictions were validated with patch-clamp studies of human HCM cardiomyocytes (*n* = 12 cells, *N* = 5 patients). Ventricular simulations were informed by typical distributions of subendocardial/transmural ischaemia as analysed in perfusion scans (*N* = 28 patients). S1-S2 pacing protocols were used to quantify arrhythmic risk for scenarios in which regions of septal obstructive hypertrophy were affected by (i) ischaemia, (ii) ischaemia and impaired repolarization, and (iii) ischaemia, impaired repolarization, and diffuse fibrosis. HCM cardiomyocytes exhibited enhanced action potential and abnormal effective refractory period shortening to ischaemic insults. Analysis of ∼75 000 re-entry induction cases revealed that the abnormal HCM cellular response enabled establishment of arrhythmia at milder ischaemia than otherwise possible in healthy myocardium, due to larger refractoriness gradients that promoted conduction block. Arrhythmias were more easily sustained in transmural than subendocardial ischaemia. Mechanisms of ischaemia–fibrosis interaction were strongly electrophysiology dependent. Fibrosis enabled asymmetric re-entry patterns and break-up into sustained ventricular tachycardia.

**Conclusion:**

HCM ventricles exhibited an increased risk to non-sustained and sustained re-entry, largely dominated by an impaired cellular response and deleterious interactions with the diffuse fibrotic substrate.

## Graphical Abstract

**Graphical Abstract:**
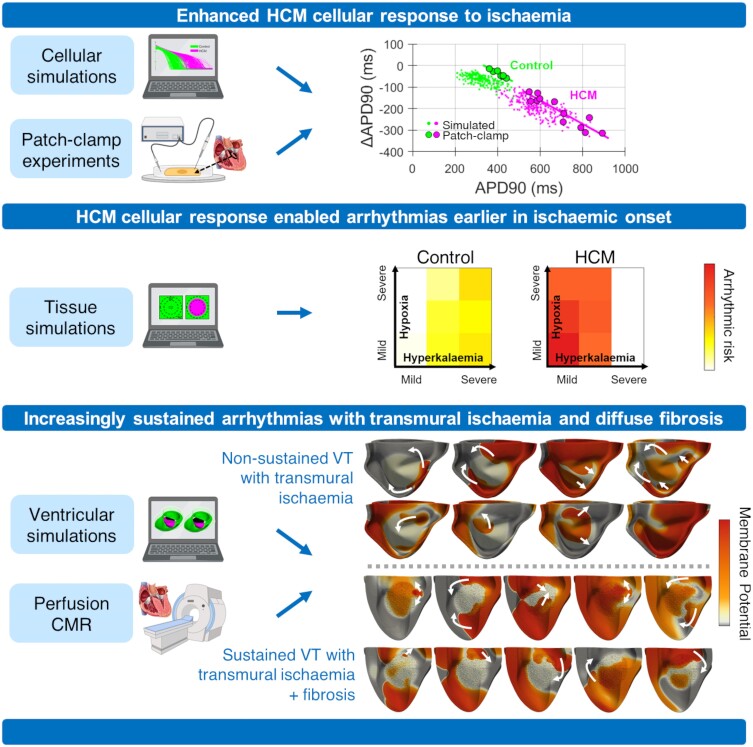

**Time of primary review: 62 days**

## 1. Introduction

Hypertrophic cardiomyopathy (HCM) is one of the most common genetic heart diseases and a leading cause of sudden cardiac death (SCD) in the young, with most SCDs being unpredicted.^1^ Acute myocardial ischaemia is acknowledged as a cause of ventricular arrhythmias in clinical guidelines^2^ and is considered a key cause of SCD in young patients without major structural remodelling.^3^ Despite ischaemia being a predictor of adverse cardiovascular events in some studies,^4^ the HCM-specific mechanisms underlying ischaemia-induced arrhythmias are not fully understood, nor how acute ischaemia interacts with other pathological features of HCM such as diffuse fibrosis and chronic ionic remodelling.

In the general population, acute myocardial ischaemia is well characterized as a substrate and trigger for lethal arrhythmias, with coronary artery disease remaining the major cause of SCD. However, in HCM ventricles, there is a tendency for ischaemia to colocalize with hypertrophy,^4^ which generally does not relate to coronary artery territories. Despite being distinct from coronary artery disease, myocardial ischaemia in HCM can still cause acute myocardial infarction,^5^ through several pro-ischaemic mechanisms including diffuse small vessel disease, energetic impairment of the sarcomere, increased muscle mass, reductions in capillary density, excessive extravascular compression, and left ventricular (LV) outflow tract obstruction.^4^

Repolarization abnormalities secondary to ionic remodelling, manifesting as action potential (AP) prolongation in regions of hypertrophy, are common in HCM and are understood to promote ectopic triggers.^6^ Yet, how the HCM-mediated ionic remodelling may modulate the ischaemic arrhythmic substrate remains unknown, despite evidence of pro-arrhythmic hypertrophy–ischaemia interactions in the general population.^7^ Repolarization impairment in HCM is of clinical importance because it is an arrhythmic substrate modifier that is detectable on the electrocardiogram^8^ and could be amenable to pharmacologic therapy.^9^

In addition, diffuse and replacement fibrosis are common in HCM in regions of hypertrophy,^10^ with extensive fibrosis and scarring identified by late gadolinium enhancement (LGE) imaging being a SCD risk factor recognized in clinical guidelines. However, as LGE is closely related to perfusion impairment, disentangling independent contributions to arrhythmic risk by ischaemia vs. fibrosis is challenging in HCM, as both factors are commonly present and overlap substantially on imaging.^4^ Moreover, investigating arrhythmia mechanisms from ischaemia and fibrosis either *in vivo* or *in vitro* becomes infeasible in HCM, not only due to scarcity in human data but also because of the absence of relevant animal models and the impossibility of performing invasive mapping protocols in this group of high-risk patients.

To overcome such limitations, in this study, a multiscale digital twin approach is exploited in synergy with clinical perfusion imaging and human cardiomyocyte patch-clamp experiments to identify disease-specific mechanisms of ischaemic arrhythmic risk in HCM and to investigate its modulation by ionic remodelling and fibrosis.^11^ The hypothesis is that, because gradients of refractoriness are common precursors of conduction block and subsequent re-entry in myocardial ischaemia,^12^ increased refractoriness heterogeneity secondary to chronic ionic remodelling in hypertrophied regions will increase vulnerability to conduction block and hence arrhythmic risk. It was further hypothesized that exacerbation of ischaemic conduction delay by diffuse fibrosis enables increasingly sustained arrhythmias during myocardial ischaemia in HCM.

## 2. Methods

### 2.1 Cellular electrophysiology response to ischaemia

The HCM cellular electrophysiological response to acute ischaemic effects was investigated using the ToR-ORd AP model, which is a computational tool used to simulate human ventricular cardiomyocyte electrophysiology,^13^ with biophysically detailed ion channel behaviours that produce validated AP and Ca^2+^ transient biomarkers. Under baseline conditions, cellular repolarization is impaired in the diseased myocardium of HCM patients due to remodelling in ion channels (primarily upregulation of late Na^+^ and downregulation of K^+^ channels) and intracellular Ca^2+^ handling, as characterized *in vitro* in surgical myectomy samples.^6^ This ionic remodelling pattern reported experimentally was introduced *in silico*, by remodelling ion channel conductances (see Supplementary material online, *Table S1*) as in previous work.^14^

**Figure 1:**
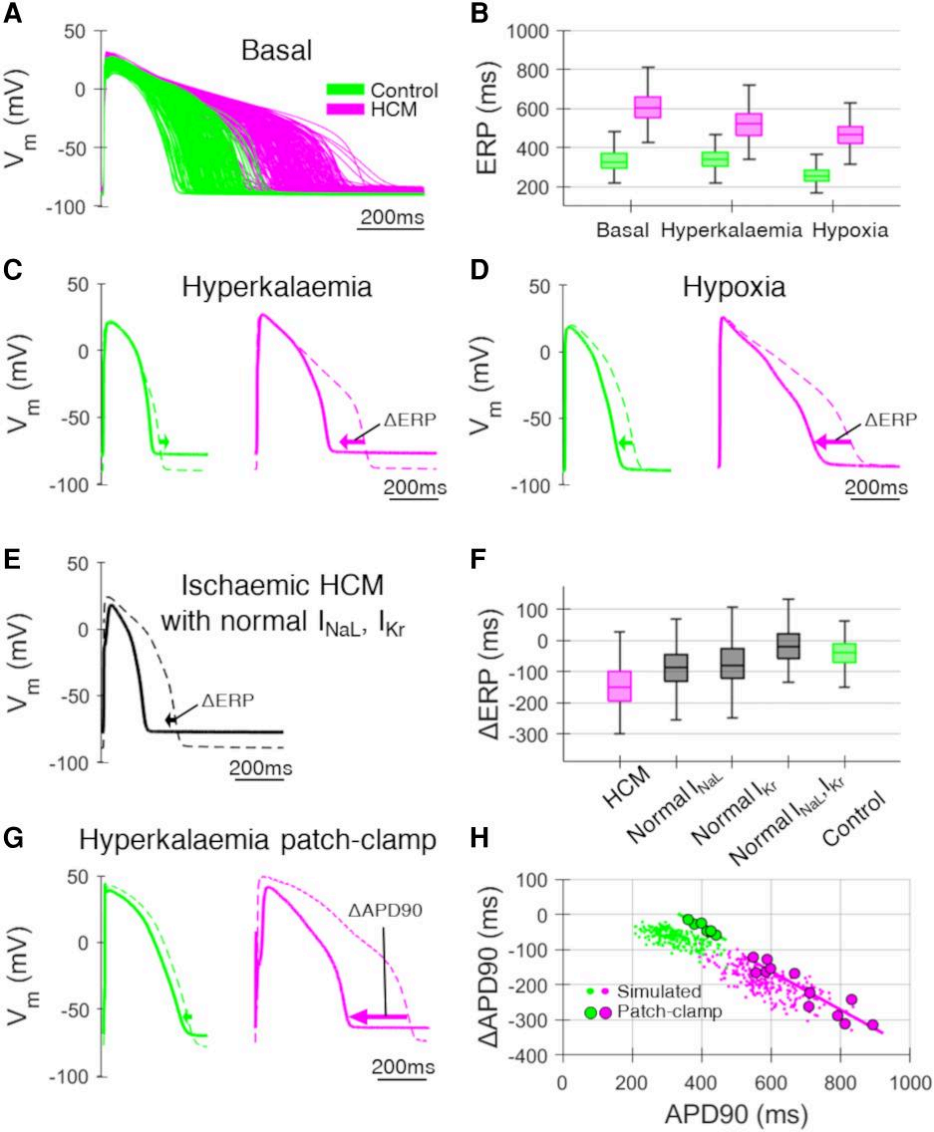
Cellular HCM response to ischaemia. (*A*) Simulated populations of non-ischaemic control (*n* = 228) and HCM (*n* = 228) human AP models. (*B*) ERPs among the populations under basal, hyperkalaemic (*K*_o_ = 8 mM), and hypoxic (*f*_KATP_ = 0.09) conditions, showing abnormal hyperkalaemic and enhanced hypoxic ERP shortening in HCM. (*C*) Representative simulated AP traces during hyperkalaemia (*K*_o_ = 8 mM; solid) compared to basal conditions (dashed). Arrows denote change in ERP due to hyperkalaemia. (*D*) Representative simulated AP traces during hypoxia (*f*_KATP_ = 0.09; solid) compared to basal conditions (dashed). Arrows denote change in ERP due to hypoxia. (*E*) Representative simulated AP traces with HCM remodelling except in *I*_NaL_ and *I*_Kr_ during ischaemia (hyperkalaemia, hypoxia, and acidosis; solid) compared to basal conditions (dashed). Arrow denotes change in ERP due to hyperkalaemia. (*F*) The difference in ERP during ischaemia with respect to basal conditions (ΔERP) among populations with full HCM ionic remodelling, HCM except *I*_NaL_ upregulation, HCM except *I*_Kr_ downregulation, HCM except both *I*_NaL_ and *I*_Kr_ remodelling, and control electrophysiology. (*G*) Representative patch-clamp human AP traces during hyperkalaemia (*K*_o_ = 9 mM; solid) compared to basal conditions (dashed) paced at 0.5 Hz (*n* = 12 HCM cells from *N* = 5 patients; *n* = 7 control cells from *N* = 3 patients). (*H*) The difference in APD90 during hyperkalaemia (*K*_o_ = 9 mM) with respect to basal conditions (ΔAPD90) vs. basal APD90 in patch-clamp experiments, compared to the simulated AP models paced at 0.5 Hz. Patch-clamp experiments confirmed the relationship between basal APD and hyperkalaemic APD decreases in control and HCM (both *P* < 0.001).

Inter-subject electrophysiological variability was considered by repeating cellular simulations for populations of cardiomyocyte AP models, where 1000 candidate AP models were initially generated by uniformly randomizing ion channel conductances in the range [−50%, +50%] of their default value, and then 228 AP models were included in the final AP population on the basis that their AP biomarkers fell within human experimental ranges.^14^ This resulted in 228 control human AP models, paired to 228 HCM human AP models (where ionic remodelling has been applied to each respective control model), as shown in *Figure 1A*.

The populations of control and HCM human AP models were identically subjected to Phase 1A ischaemia, the early reversible stage of infarction characterized by high arrhythmic risk. Phase 1A ischaemia was modelled considering an increased extracellular K^+^ concentration (*K*_o_; hyperkalaemia), ATP-sensitive K^+^ channel (*I*_KATP_) activation by a scaling factor *f*_KATP_ (hypoxia), and pH-induced impairment of L-type Ca^2+^ and Na^+^ channels (acidosis), as in previous work.^12,15–17^ The populations of AP models were subjected to these individual effects of ischaemia and to its combined effects. Hyperkalaemic severity (modelled for *K*_o_ = [7, 8, 9] mM) was informed by measurements of *K*_o_ from *in situ* pigs, in which peak *K*_o_ of 7–9 mM was reported in the central ischaemic zone during 3–10 min of initial LAD occlusion.^18^ Activation of *I_KATP_*, reported experimentally during Phase 1A ischaemia,^19–21^ was informed by previous comparisons between computational electrophysiology models and experimental measurements in ischaemic human ventricles of AP duration (APD)^22^ and fibrillation dominant frequency behaviour.^23^ These analyses found reasonable agreement with experimental recordings for activation fractions equivalent to *f*_KATP_ = [0.0, 0.038, 0.075, 0.11, 0.15]^22^ and *f*_KATP_ = [0.0030, 0.023, 0.35],^23^ where differences in maximal channel conductance were adjusted for. Activation of *I*_KATP_ was modelled for *f*_KATP_ = [0.0, 0.030, 0.060, 0.090]. Acidotic impairment of L-type Ca^2+^ and Na^+^ channels was introduced as a fixed 25% reduction in the maximal channel conductances.^16,24–26^ In tissue simulations, a hyperkalaemia/hypoxia sensitivity analysis was performed rather than explicitly modelling different time points in acute ischaemia,^17^ because there may be differences in K^+^ accumulation rate in HCM^21^ and whether widespread impairment of K^+^ channels extends to *I*_KATP_ channels is unknown.^6^

AP models were paced for 100 beats with a cycle length of 1000 ms. To quantify the cellular response to ischaemia, AP biomarkers including APD and effective refractory period (ERP; ERP methodology detailed in Supplementary material online, *Section S1.2*) were measured under basal and various acute ischaemic conditions, for the control and HCM AP populations.

### 2.2 Characterizing arrhythmic risk during ischaemia in HCM

Simulations were extended to 2D tissue and 3D biventricular human models of regional acute myocardial ischaemia to characterize the contribution of ionic remodelling and diffuse fibrosis to ischaemic arrhythmic risk. To test the hypothesis that HCM ionic remodelling increases arrhythmic risk during ischaemia, scenarios were considered where (i) regional acute myocardial ischaemia colocalizes with baseline impaired repolarization due to HCM ionic remodelling and (ii) regional acute myocardial ischaemia occurs under control electrophysiology. To test the hypothesis that fibrosis modulates arrhythmic risk during ischaemia in HCM, simulations were conducted where (iii) regional acute myocardial ischaemia colocalizes with baseline impaired repolarization and diffuse fibrosis and compared with scenario (i).

#### 2.2.1 Tissue and biventricular models of ischaemia and fibrosis

Regional acute myocardial ischaemia was modelled in human 2D and 3D tissues including the (i) core ischaemic zone (CZ), (ii) lateral border zone (BZ), and (iii) endocardial BZ.^15^ Ischaemia was modelled as circular/spherical with a diameter of 5 cm (including CZ and BZ),^12,15,16,27^ consistent with reported concentric perfusion defects in HCM,^28^ which are frequently >3 cm in extent.^29^ The lateral BZ was modelled as 0.5 cm in width, and the endocardial BZ was modelled as 0.06 cm in width.^15,30,31^ In 3D, both subendocardial and transmural ischaemia were modelled as reported clinically,^4^ where subendocardial and transmural ischaemia were considered to affect 50 and 100% of the LV wall transmurally, respectively. Subendocardial and transmural ischaemia corresponded to 11 and 33% of LV volume affected, respectively, comparable to the reported 2–35% LV range of perfusion defect sizes reported in HCM.^32^

Myocardial fibrosis was modelled by setting a fraction of mesh elements as unexcitable, assigned stochastically using a uniform probability density function. Arrhythmic risk in fibrotic HCM tissue was measured for 10, 20, and 30% fibrotic densities, for 10 randomized distributions at each value. Arrhythmic risk in fibrotic human HCM ventricles was measured at 30% fibrotic density, for one randomized distribution.

Human tissue simulations used a square domain. Simulations in tissue were repeated using 50 different AP models randomly sampled from the AP populations to account for inter-subject variability in disease expression.

Human biventricular simulations used a hexahedral mesh derived from an HCM patient with septal hypertrophy. This incorporated transmural heterogeneity (70% endocardial, 30% epicardial cells) and human conduction velocities.^8^ Apex-to-base gradients in APD were further included by rescaling the maximal *I*_Ks_ conductance in the range [0.2, 5], scaled with an exponential gradient in distance from a point defined at the apex.^8^ Fibre, sheet, and normal directions and human conductivity anisotropy were also modelled.^8^ All simulations were performed using the MonoAlg3D cardiac electrophysiology simulator^33^ coupled to the ToR-ORd human ventricular AP model^13^ and used a spatial discretization of 250 or 300 μm.

#### 2.2.2 Ectopic S1-S2 pacing protocol

Arrhythmic risk was quantified in tissue and biventricular models using an ectopic S1-S2 pacing protocol to attempt to induce arrhythmia in each disease scenario. After AP model stabilization, the S1-S2 protocol consisted of two sinus rhythm beats (S1) at a cycle length of 1000 ms, followed by an ectopic stimulus (S2). For each arrhythmic substrate, whether arrhythmia can be induced, and the duration of the resulting arrhythmias, gauges the arrhythmic risk.

In tissue models, the S1 stimulus was simulated as a planar wavefront applied to an edge of the square domain. In the biventricular model, a human physiological activation sequence was used as S1, as in previous work.^8,34^ Briefly, seven root nodes were defined on the endocardium, based on sites of earliest activation resulting from the human trifascicular conduction system. Each endocardial surface node was then assigned a stimulus time proportional to its distance to its closest root node, as computed with Dijkstra’s algorithm.

In tissue and biventricular models, the S2 stimulus was applied in the lateral BZ of the ischaemic region, approximating experiments in which premature beats emerged from the NZ directly adjacent to the BZ and induced re-entries.^35^ In tissue models, S2 was applied mid-BZ proximal to the side of S1. In biventricular models, to account for the non-planar S1 activation, experiments were repeated for six different S2 positions to sufficiently cover the ischaemic BZ in the apico–basal and anterior–posterior planes, applied endocardially mid-BZ and equispaced around the region of ischaemia.

Experiments were repeated in tissue and biventricular models for a range of S1-S2 time intervals. In tissue models, S1-S2 time intervals were varied in 5 ms increments in the range [ERP_NZ_ − 20, ERP_CZ_ + 120] where ERP_NZ_ and ERP_CZ_ are the ERPs of the normal and CZs of each AP model. In some AP models, the range of S1-S2 intervals was extended to ensure that a minimum of 10 S1-S2 coupling intervals were simulated. In biventricular models, a minimum of 10 S1-S2 intervals were considered, in 5 ms increments following the minimum S1-S2 interval for which S2 propagated.^12^

### 2.3 Patch-clamp

Briefly, viable single myocytes (*n* = 12) were obtained from septal samples from HCM patients (*N* = 5) who underwent septal myectomy and were compared to control myocytes (*n* = 7) from non-failing non-hypertrophic aortic stenosis patients (*N* = 3) with bulging septa who underwent myectomy. Of note, these control cells had slightly longer basal APD than totally healthy myocytes. APs were measured in these myocytes at stimulation frequencies of 0.2 and 0.5 Hz, for extracellular K^+^ concentrations of *K*_o_ = 5 mM and *K*_o_ = 9 mM. The studies were approved by the local Ethics Committee, and written informed consent was obtained from all participants. This investigation conforms to the principles outlined in the Declaration of Helsinki (see Supplementary material online, *Section S1.3*, for full patch-clamp experimental methods).

### 2.4 Perfusion imaging

Twenty-eight HCM patients (see Supplementary material online, *Table S2*) were prospectively enrolled from the inherited cardiac condition (ICC) clinic at the John Radcliffe Hospital in Oxford, UK. All participants provided informed written consent. The CMR study was approved by the National Research Ethics Committee (REC ref 12/LO/1979).

After appropriate clinical screening, HCM patients underwent adenosine vasodilator stress cardiac magnetic resonance (CMR) imaging at 3 Tesla (Trio, Siemens Healthcare, Erlangen, Germany). The CMR protocol included adenosine vasodilator stress and rest first-pass perfusion imaging to assess for perfusion abnormalities. Adenosine vasodilator stress perfusion was performed at a standard 140 μg/kg/min, and in non-responders, it was up-titrated as previously described.^36^ For perfusion analysis, myocardial perfusion reserve index (MPRI) was measured from stress and rest signal intensity curves as previously described.^37^

### 2.5 Statistical methods

Data are expressed as mean ± SD; *t*-tests were applied to compare MPRI measurements in each LV segment between patients with and without a history of ventricular tachycardia using the scipy.stats Python library. In patch-clamp experiments, the relationship between basal APD and the hyperkalaemic change in APD was assessed using the MATLAB corrcoef() function. Values of *P* < 0.05 were considered statistically significant.

## 3. Results

### 3.1 Cellular electrophysiology response to ischaemia in HCM

The populations of human AP models (228 controls paired to 228 HCMs) under basal conditions are shown in *Figure 1A*. Consistent with experimental evidence,^6^ the HCM AP models were characterized by markedly prolonged AP prolongation and increased ERP, as shown in *Figure 1B*. Hyperkalaemia caused APD decreases, post-repolarization refractoriness, and made resting membrane potentials less negative in both populations (*Figure 1C*), as is typical of hyperkalaemia.^22^ AP shortening typical of hypoxia also occurred in both populations (*Figure 1D*). Acidosis led to an expected 10% reduction in conduction velocity in both control and HCM AP models (see Supplementary material online, *Figure S1*).^24^

However, marked differences were observed in the response of ERP to ischaemia between control and HCM AP models. In control models, ERP increased slightly during hyperkalaemia from 330 ± 60 to 340 ± 50 ms while instead significantly decreased in HCM models from 600 ± 80 to 520 ± 80 ms (*Figure 1B* and *C*). In control models, ERP decreased during hypoxia to 260 ± 40 ms whereas it decreased by a larger extent in HCM models to 460 ± 60 ms (*Figure 1B* and *D*).

Components of the HCM ionic remodelling were selectively removed to identify the cause of these abnormalities, which showed that remodelling of the late sodium (*I*_NaL_) and the rapid delayed rectifier K^+^ (*I*_Kr_) currents were responsible (*Figure 1E* and *F*). Specifically, resting membrane potentials becoming less negative during hyperkalaemia led to reduced late sodium current availability, due to the voltage dependence of late sodium inactivation gating (see Supplementary material online, *Figure S2*). This effect was magnified in HCM where late sodium channels are overexpressed, leading to enhanced reductions in APD during ischaemia relative to control cardiomyocytes.

The degree of hyperkalaemic AP shortening in HCM (*Figure 1C*) underlying the hyperkalaemic ERP decreases predicted by the simulation study was experimentally confirmed in patch-clamp experiments of human HCM cardiomyocytes (*Figure 1G* and *H*). An increase in extracellular K^+^ concentration from *K*_o_ = 5 mM to *K*_o_ = 9 mM led to an APD decrease of 210 ± 70 ms experimentally in human HCM cardiomyocytes, comparable to the decrease of 200 ± 40 ms identified in simulations. In patch-clamp experiments, longer basal APDs were associated with greater hyperkalaemic APD shortening in control and HCM (*P* < 0.001 both; *Figure 1H*). Although the control cells were from aortic stenosis patients with slightly longer basal APDs than simulated control myocytes, the degree of hyperkalaemic APD shortening was consistent between simulations and patch-clamp experiments.

The response of HCM-remodelled cardiomyocytes to ischaemia was therefore characterized by (i) an opposite ERP response to hyperkalaemia (shortening) and (ii) enhanced AP shortening by hypoxia and hyperkalaemia, when compared with control cardiomyocytes.

Effects on Ca^2+^ transient biomarkers are further shown in Supplementary material online, *Figure S3*, where myocardial ischaemia led to similar changes in both populations. Of note, Ca^2+^ transient durations in HCM remained prolonged at 570 ± 30 ms compared to controls at 440 ± 50 ms during ischaemia. This contrasts with the significant ischaemic APD decreases observed in HCM models, which may have implications for cellular arrhythmias in HCM. The sensitivity of AP biomarkers to additional ischaemic effects in *I*_NaK_, *I*_NaL_, and *I*_NCX_ was investigated in Supplementary material online, *Figure S4*.

### 3.2 Arrhythmic risk characterized in tissue

The cellular investigations motivated an in-tissue sensitivity analysis to investigate how the degree of hypoxia/hyperkalaemia modulates arrhythmic risk differently in control and HCM-remodelled myocardium. The degree of acidosis was fixed in the sensitivity analysis because it had negligible effects on refractoriness and identical effects on conduction velocities between control and HCM AP models (see Supplementary material online, *Figure S1*).

#### 3.2.1 Re-entry emerges earlier in the onset of ischaemia in HCM tissue

**Figure 2:**
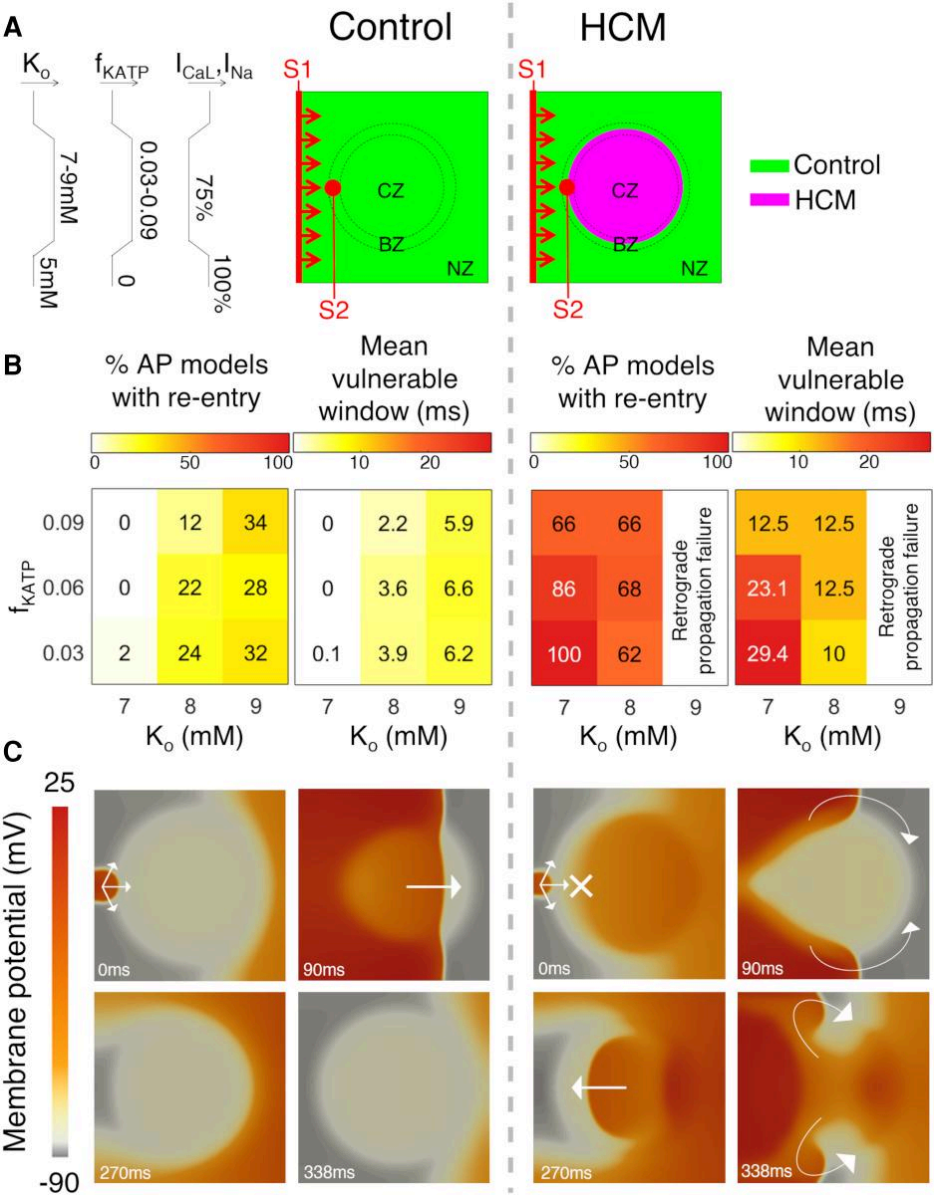
In-tissue characterization of HCM ischaemic arrhythmic risk. (*A*) Schematics of in-tissue acute myocardial ischaemia domains, with control electrophysiology (left) and regional HCM electrophysiology (right), for which arrhythmic risk was measured in 50 randomly sampled AP models. Domains consist of a CZ subject uniformly to the full extent of ischaemia, a BZ where ischaemic parameters (*K*_o_, *f*_KATP_, and *I*_CaL_, *I*_Na_ inhibition) are linearly varied towards baseline values away from the CZ (left-hand side diagrams), and a normal zone (NZ) unaffected by ischaemia. The positions of sinus rhythm activation (S1) and the ectopic stimulus (S2) are denoted. (*B*) Summary statistics of arrhythmic risk over the hypoxia/hyperkalaemia parameter space for the 50 AP models in tissue, as characterized using ectopic S1-S2 pacing protocols. (*C*) Representative in-tissue simulations at *K*_o_ = 7 mM and *f*_KATP_ = 0.06 following application of S2 demonstrate how conduction block is infrequent in ischaemic control myocardium (left), but common in ischaemic HCM myocardium (right) due to repolarization impairment. Subsequent re-entries occur in HCM. The circular ischaemic region has visibly less negative resting membrane potential. Arrows and crosses indicate direction of wavefront propagation and locations of conduction block, respectively. Time elapsed (ms) following S2 is denoted in each frame. *K*_o_, extracellular K^+^ concentration (mM); f_KATP_, fraction of activated K-ATP channels; *I*_CaL_, *I*_Na_, L-type Ca^2+^ and fast Na^+^ currents.

Arrhythmic risk studies were performed for 900 in-tissue scenarios, spread across 50 human control and HCM AP models randomly sampled from the populations, for 9 parameter combinations of hyperkalaemia and hypoxia. This consisted of ectopic S1-S2 protocols applied to tissue models of acute regional myocardial ischaemia (*Figure 2A*) for a range of coupling intervals, totalling 28 040 simulations of re-entry inducibility. Analysis of the arrhythmic risk studies highlighted that ischaemia-induced re-entry can emerge at a much lesser ischaemic extent under conditions of HCM ionic remodelling than in non-diseased myocardium.

The summary statistics of arrhythmic risk in *Figure 2B* show that only 0–2% of control models enabled re-entry at low hyperkalaemic extent (*K*_o_ = 7 mM) compared to 66–100% of models affected by HCM ionic remodelling. Moreover, the range of S1-S2 time intervals at which re-entry occurred (the vulnerable window) at low hyperkalaemic extent was also markedly greater in HCM, ranging between 12.5 and 29.4 ms compared to 0–0.1 ms in controls. These considerations also held at intermediate hyperkalaemic extent (*K*_o_ = 8 mM). Ischaemia in control tissue models only became relatively pro-arrhythmic at high hyperkalaemic concentrations (*K*_o_ = 9 mM), for which the HCM-remodelled region failed to support retrograde propagation.

Differences in re-entry inducibility are explained by differences in ERP between ischaemic control and HCM tissue (*Figure 1B*) and the dependence that figure-of-eight re-entry has on ERP gradients. Figure-of-eight re-entry requires that (i) S2 propagates in the NZ; (ii) S2 is blocked at the CZ; (iii) retrograde propagation occurs in the CZ; and (iv) retrograde propagation can re-excite the NZ.

Conditions (i) and (ii) approximately require that ERP_CZ_ > ERP_NZ_, a condition which was already satisfied by the regional HCM ionic remodelling without ischaemia (*Figures 1B* and *2C*). The contribution of ischaemia to arrhythmic risk in 2D HCM models is that, through shortening of ERP_CZ_ and conduction velocity, conditions (iii) and (iv) can be satisfied in an ischaemic region of realistic size. Conversely, in control models, the condition that ERP_CZ_ > ERP_NZ_ requires a threshold extent of hyperkalaemia above which the ERP is prolonged (and above which this outweighs hypoxic ERP shortening). Specifically, at *K*_o_ = 7 mM, there are sufficient reductions in the conduction velocity and ERP_CZ_ to enable re-entry in HCM models, whereas in control models, ERP_CZ_ is only sufficiently prolonged beyond a threshold *K*_o_ = 8 mM.

It is notable that, although arrhythmic risk decreased as ischaemia progressed from mild to severe extents of hyperkalaemia and hypoxia in HCM models (*Figure 2B*), arrhythmic risk for *K*_o_ = 7–8 mM was significantly greater than that under non-ischaemic conditions (where only 16% of models enabled re-entry). Even under control electrophysiology, a decrease in arrhythmic risk for progression to severe ischaemic parameters is frequently observed in computational models,^16,17^ and the high arrhythmic risk occurring at an intermediate degree of ischaemia^17^ may also apply to HCM. This is because for hyperkalaemia *K*_o_ > 10 mM, total conduction block often occurs.^24^ Indeed, the failure of re-entries to emerge in HCM tissue models at high hyperkalaemic extent (*K*_o_ = 9 mM) was typically caused by retrograde propagation failure. Ionic remodelling in HCM includes Na^+^/K^+^ pump impairment,^14^ which made resting membrane potentials even less negative and constrained sodium current availability for AP propagation (see Supplementary material online, *Figure S5*). Retrograde propagation typically involved thin wavefronts that were unable to overcome this effect.

The dependence of arrhythmic risk during myocardial ischaemia in-tissue on the extent of regional HCM baseline repolarization impairment was further analysed in Supplementary material online, *Figure S6*. These findings suggest that, in the early stages of HCM, arrhythmic risk may increase as ionic remodelling develops, but that in the later stages, marked ERP prolongation may preclude re-entries. The sensitivity of in-tissue arrhythmic risk to additional ischaemic effects in *I*_NaK_, *I*_NaL_, and *I*_NCX_ was investigated in Supplementary material online, *Figure S7*, showing that these components had minor consequences for arrhythmic risk. In a subset of in-tissue simulations, sensitivity of the in-tissue arrhythmic risk to hypoxic BZ gradient steepness and spatial resolution was investigated in Supplementary material online, *Figures S8* and *S9*, respectively, showing only modest sensitivity to these factors.

#### 3.2.2 Multiple mechanisms of in-tissue ischaemia–fibrosis interaction

Arrhythmic risk modulation by ischaemia–fibrosis interactions was investigated for 450 HCM tissue scenarios, spread across 15 human HCM AP models, for the 3 fibrotic extents in 10, 20, and 30%, where randomized distributions of fibrosis were sampled 10 times at each fibrotic density. Ectopic stimulus protocols were applied to tissue models of acute regional myocardial ischaemia with diffuse fibrosis, for a range of coupling intervals totalling 16 950 simulations of re-entry inducibility. Informed by our results on ischaemic burden in HCM, episodes of ischaemia were simulated using the hyperkalaemia and hypoxia parameters *K*_o_ = 7 mM and *f*_KATP_ = 0.06. The 15 human HCM AP models were chosen from the HCM population such as to include a set of AP models exhibiting variability in arrhythmic risk in the prior non-fibrotic scenarios. Specifically, no re-entry vs. induced re-entry emerged in 8 and 7 AP models, respectively, in the non-fibrotic case.

**Figure 3:**
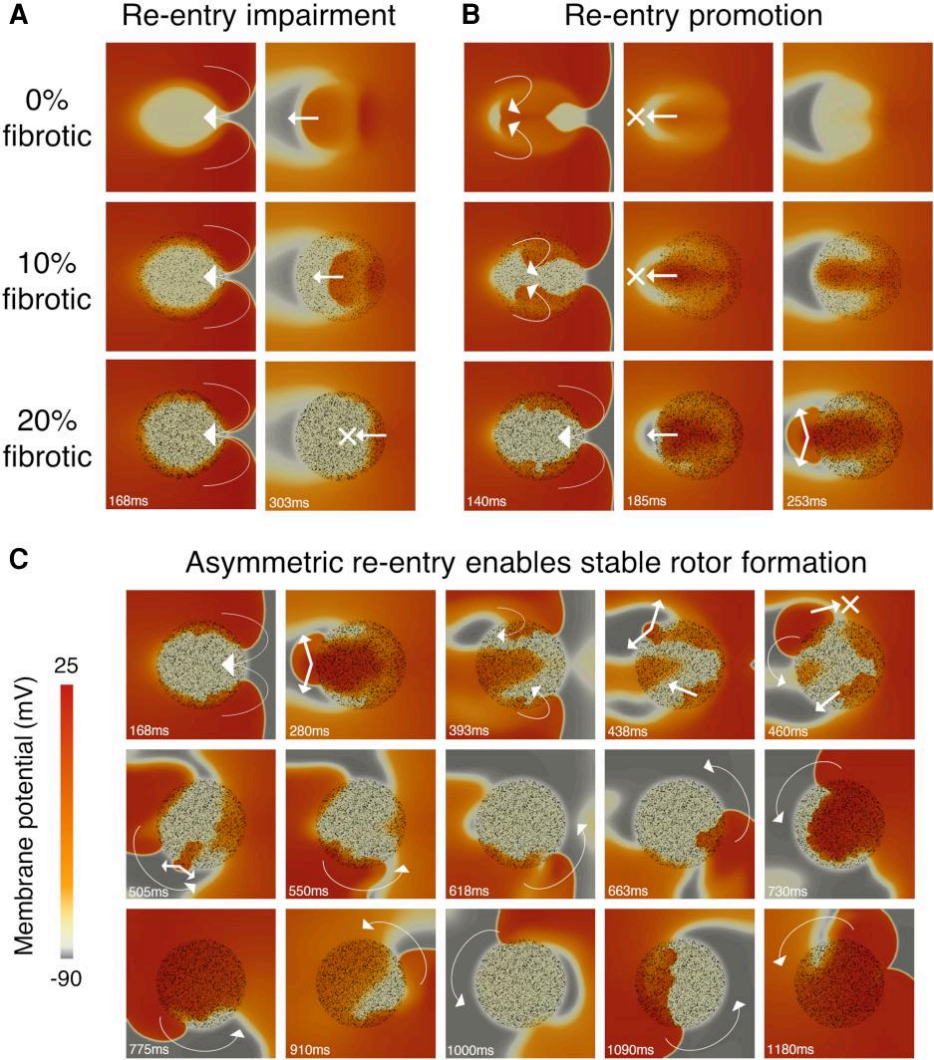
Diffuse fibrosis modulates arrhythmic risk during ischaemia in HCM. Representative in-tissue simulations following application of S2 demonstrate how the presence of diffuse fibrosis affects re-entry inducibility in ischaemic HCM myocardium, through three different mechanisms. Conduction impairment at increased fibrosis density leads to (*A*) retrograde propagation block at the CZ, preventing re-entrant cycles, or (*B*) retrograde propagation delay at the CZ, enabling re-entrant cycles by allowing NZ to recover excitability. (*C*) The fibrotic microstructure leads to asymmetric retrograde propagation, such that the re-entrant wavefront interacts with the refractory wavetail and forms a stable rotor, anchored to the ischaemic–fibrotic region. Arrows and crosses indicate direction of wavefront propagation and locations of conduction block, respectively. Time elapsed (ms) following S2 is denoted in each frame.

Analysis of in-tissue arrhythmic risk simulations identified that mechanisms of ischaemia–fibrosis interaction are strongly dependent on the underlying electrophysiology. Among some AP models, fibrosis had a net antiarrhythmic effect (*Figure 3A*) by interrupting retrograde propagation through slowing or impairing conduction in the core zone. In the example shown in *Figure 3A*, retrograde propagation was able to complete with no fibrosis (0%) or mild fibrotic infiltration (10%), but with further impaired conduction from greater fibrosis content (20%), total conduction block occurred. In other AP models, fibrosis had a pro-arrhythmic effect by delaying core zone conduction enough for timely normal zone recovery. As shown in *Figure 3B*, retrograde propagation with no fibrosis (0%) or mild fibrotic infiltration (10%) allowed re-excitation of the core zone shortly after the ectopic stimulus. With greater fibrosis content (20%), core zone conduction was impaired such that retrograde propagation was delayed enough for the normal zone to recover excitability, which allowed re-entry.

Some patterns of fibrosis enabled re-entrant wavefronts to emerge asymmetrically, such that they interacted with the wavetail of previous re-entrant cycles, promoting sustained break-up (*Figure 3C*). In this case, clockwise propagation was blocked by a refractory wavetail, while anticlockwise propagation persisted. This formed a stable rotor anchored to the ischaemic–fibrotic region. These results suggest that breaking the symmetry of figure-of-eight re-entries is one mechanism by which fibrosis increases arrhythmia sustainability in HCM. The circuit length of a single asymmetric rotor is greater than that of each of two symmetric re-entries in a figure-of-eight; hence, they are less easily interrupted by fluctuations in cycle-to-cycle activation and ERP.

AP models in which fibrosis increased arrhythmic risk during ischaemia tended to have a higher Na^+^ conductance and lower ERP in the ischaemic core zone of affected HCM myocardium. This is because, with higher Na^+^ conductance, fibrosis delayed conduction but was unable to block retrograde propagation entirely. In these models, the ERP in the core zone was too low to block conduction long enough for ERP in the external normal zone to recover excitability (*Figure 3B*) unless fibrosis was present to delay retrograde propagation. When fibrosis promoted re-entry, it was more likely to be sustained because ERP in the core zone was short in these cases. These findings suggest that fibrosis may play a pro-arrhythmic role in less diseased HCM states (lesser degree of ionic remodelling and healthy conduction), but antiarrhythmic later (greater ionic remodelling and impaired conduction). Interactions between the density of fibrosis and the ERP during hypoxia have been explored in previous computational modelling,^38^ and the present study shows that similar effects may be relevant in HCM due to further ERP modulation by ionic remodelling.

### 3.3 Arrhythmic risk characterized in HCM ventricles

#### 3.3.1 Clinically informed ischaemia and fibrosis distributions

Biventricular simulations of acute myocardial ischaemia in HCM were informed by segmental mid-slice LV measurements of MPRI from *n* = 28 HCM mutation carriers with LV hypertrophy, acquired with stress perfusion CMR imaging.

**Figure 4:**
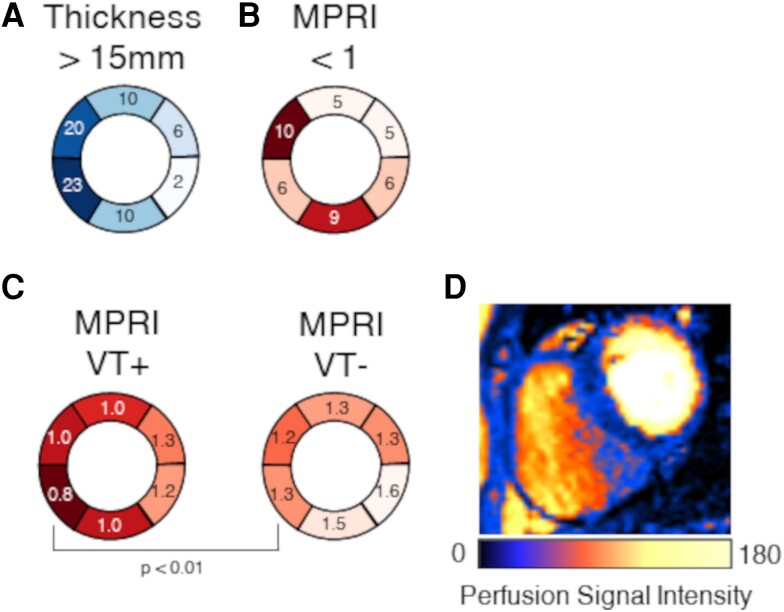
Clinical perfusion imaging data from HCM patients. (*A*) Number of segments with wall thickness >15 mm, demonstrating predominant septal involvement of hypertrophy. (*B*) Number of segments with MPRI < 1, showing that perfusion impairment is diffuse in HCM. (*C*) Comparison of MPRI between patients with and without a history of ventricular tachycardia (*n* = 5, 23 respectively), where an association was observed between inferoseptal perfusion impairment and ventricular tachycardia (*P* < 0.01). (*D*) Colour perfusion scan showing large septal perfusion defects in an HCM patient. MPRI, myocardial perfusion reserve index; VT, ventricular tachycardia.

In this cohort, hypertrophy was primarily septal (*Figure 4A*), whereas the distribution of severe impairment of the MPRI (MPRI < 1) was highly variable (*Figure 4B*), reflecting diffuse small vessel disease in HCM. Although a diffuse phenomenon, the most frequently affected segment with severe MPRI impairment was anteroseptal (*Figure 4B*). Patients with ventricular tachycardia had lower inferoseptal MPRI compared to those without (0.8 ± 0.2 vs. 1.3 ± 0.4, *P* < 0.01), as in *Figure 4C*. As ionic remodelling and LGE in HCM are hypertrophy associated,^4,6,8^ combinations of repolarization impairment, ischaemia, and fibrosis are most likely to occur in the septum; hence, all combinations of disease factors were modelled as septal.

Modelling ischaemia as septal is reinforced by the evidence that septal ischaemia in HCM is particularly common in previous works.^39–41^ Specifically, visual examples of large perfusion defects involving the septum of HCM patients can be seen in these other studies.^40,42^ Perfusion impairment is associated with hypertrophy,^4^ further motivating the inclusion of ischaemia in the region of septal hypertrophy in our reconstruction of human HCM ventricles.

#### 3.3.2 Transmural extent of ischaemia modulates arrhythmic risk in HCM ventricles

Arrhythmic risk simulations in human ventricles were conducted in nine scenarios, spread across subendocardial and transmural ischaemia for control and HCM electrophysiology. Transmurally ischaemic HCM scenarios were further simulated under the effects of mid-wall and transmural fibrosis. Ectopic stimulus protocols were applied to these ventricular scenarios for six ectopic positions at 10 coupling intervals, for a total of 540 simulations of re-entry inducibility.

**Figure 5:**
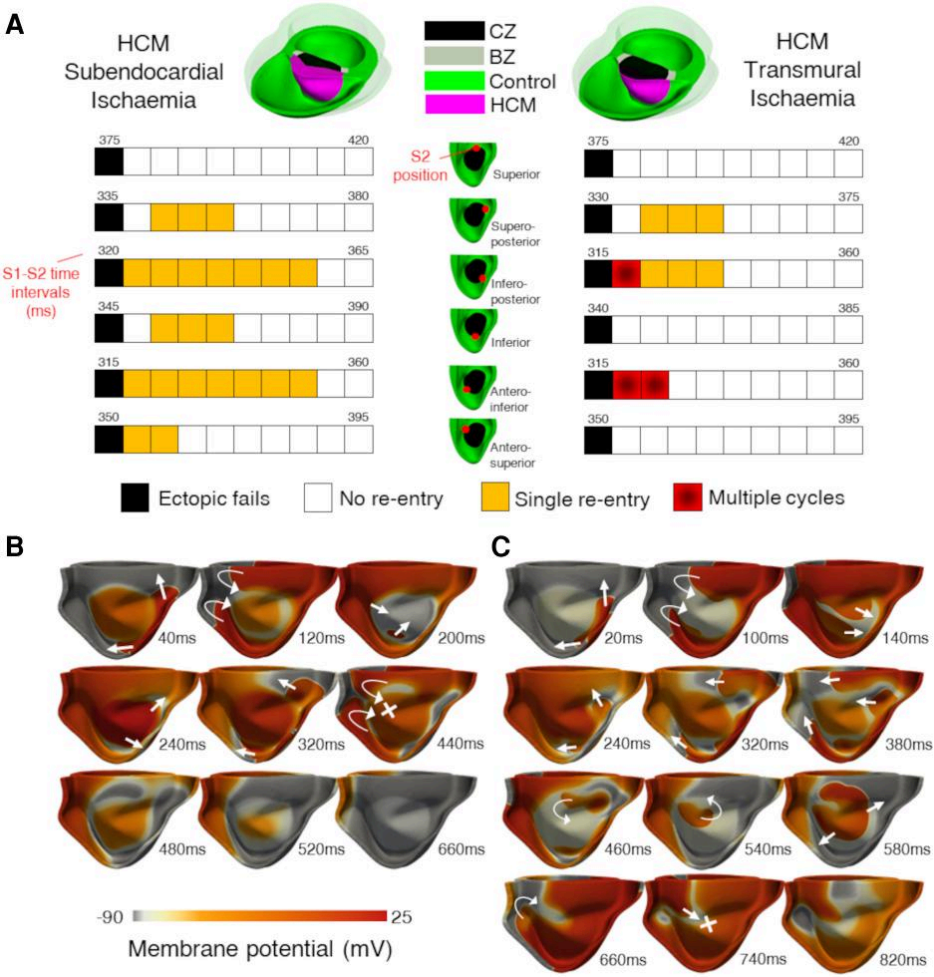
Transmural extent of ischaemia modulates arrhythmic risk in HCM. (*A*) Outcomes (either ectopic failure, no re-entry induced, single re-entry, or multiple cycles of re-entry) of the S1-S2 pacing protocol applied to the ischaemic biventricular HCM mesh, for the range of S1-S2 time intervals tested, with the minimum and maximum S1-S2 time denoted for each of the six ectopic positions. Subendocardial ischaemia (left) enabled wider windows of vulnerability to single re-entries, whereas transmural ischaemia (right) promoted increasingly sustained arrhythmia in HCM. At this low extent of ischaemia, re-entry was only inducible with regional HCM repolarization impairment. (*B*) Representative single cycle of re-entry in subendocardial ischaemia following the application of S2, compared with (*C*) multiple cycles of re-entry in transmural ischaemia, as viewed from the right ventricular cavity. Arrows and crosses indicate direction of wavefront propagation and locations of conduction block, respectively. Time elapsed (ms) following S2 is denoted in each frame.

Extending upon the in-tissue investigations in *Figure 2*, arrhythmia inducibility studies in anatomical reconstructions of the human HCM ventricles identified the transmural extent of ischaemia as a key determinant of arrhythmia sustainability. Vulnerable windows to re-entry in subendocardially and transmurally ischaemic HCM biventricular models are shown in *Figure 5A*, showing how non-sustained and sustained re-entries may be induced with appropriately timed S1-S2 coupling intervals, for six ectopic stimulus positions sufficiently covering the ischaemic BZ. Multiple cycles of re-entry only emerged in full transmural ischaemia, whereas only single re-entries were observed in subendocardial ischaemia. This is because prolonged repolarization in the region of HCM ionic remodelling unaffected by subendocardial ischaemia (right ventricle septal side) typically interrupted further re-entrant cycles (*Figure 5B*), whereas transmural ischaemia led to ERP shortening in this region that enabled further excitation and re-entry formation (*Figure 5C*). Conversely, subendocardial ischaemia manifested a greater inducibility of single re-entries. This was because the slow repolarizing non-ischaemic segment with ionic remodelling adjacent to the ischaemic subendocardium caused conduction block to occur at a wider range of S1-S2 coupling intervals. However, the region with ionic remodelling was unable to sustain re-entry due to its long ERP. This exemplifies competing effects of increased ERP on arrhythmic risk (further explored in Supplementary material online, *Figure S6*).

Moreover, comparisons of arrhythmic risk simulations in ischaemic HCM ventricles with control ventricles identified that at a mild ischaemic extent (*K*_o_ = 7 mM and *f*_KATP_ = 0.06), arrhythmia was only inducible in ventricles affected by HCM ionic remodelling. This corroborates the in-tissue findings, because again ERP was not prolonged sufficiently by ischaemia to produce conduction block unless ionic remodelling was present, even in the 3D human biventricular anatomy.

#### 3.3.3 Multiple types of fibrosis–ischaemia interaction in HCM ventricles

**Figure 6:**
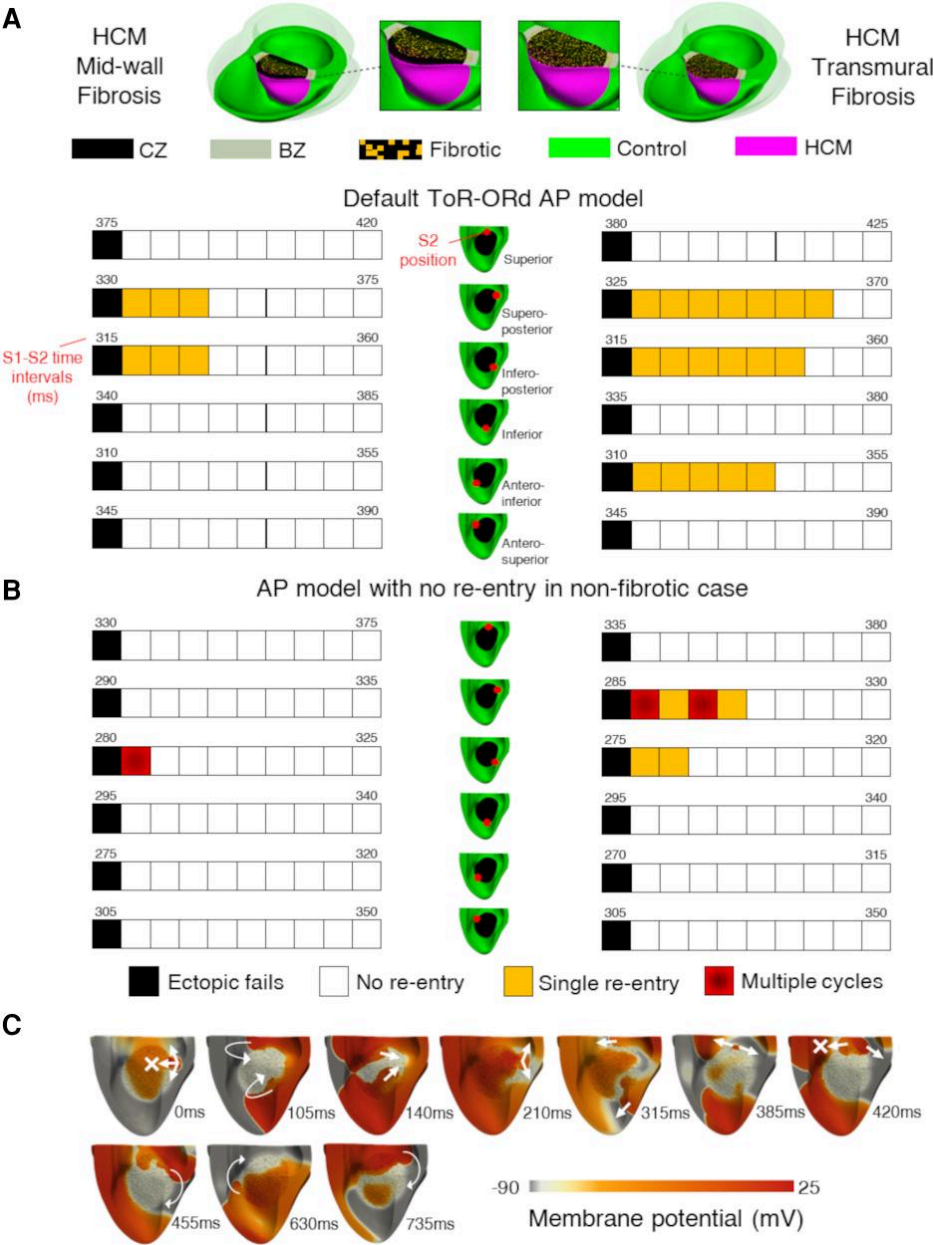
Effect of fibrosis on arrhythmic risk during ischaemia is electrophysiology dependent in HCM. Outcomes (either ectopic failure, no re-entry induced, single re-entry, or multiple cycles of re-entry) of the S1-S2 pacing protocol applied to the transmurally ischaemic biventricular HCM mesh with mid-wall (left) and transmural (right) diffuse fibrosis, for the range of S1-S2 time intervals tested, with the minimum and maximum S1-S2 time denoted for each of the six ectopic positions. (*A*) When using the default ToR-ORd AP model, multiple re-entrant cycles that were previously possible in the non-fibrotic case were interrupted by both mid-wall and transmural fibrosis by bidirectional conduction block. (*B*) In an alternative AP model from the populations, for which re-entry was not inducible under non-fibrotic conditions, mid-wall and transmural fibrosis enabled sustained re-entries to be induced by delaying conduction. (*C*) Representative sustained re-entry formation following the application of S2 for the alternative AP model in the transmurally ischaemic and fibrotic biventricular HCM mesh, where fibrosis forces a re-entry to emerge asymmetrically, which sets up a large sustained rotor anchored to the diseased myocardium. Arrows and crosses indicate direction of wavefront propagation and locations of conduction block, respectively. Time elapsed (ms) following S2 is denoted in each frame.

The dependence of fibrosis–ischaemia interaction on the underlying HCM cellular electrophysiology, as identified in tissue (*Figure 3*), is further exemplified in the transmurally ischaemic HCM ventricles (*Figure 6A* and *B*), where arrhythmic risk was investigated with contributions from mid-wall and transmural fibrosis in HCM ventricles. To facilitate comparison with non-fibrotic simulations, *Figure 6A* uses the same AP model used in *Figure 5*. To compare with the ischaemia–fibrosis interactions identified in tissue, *Figure 6B* uses the same AP model as in *Figure 3C*. To characterize the effect of increasing transmural extents of fibrosis, diffuse fibrosis was simulated in mid-wall and transmural distributions.

In the first scenario (*Figure 6A*), both mid-wall and transmural fibrosis were sufficient to interrupt the multiple cycles of re-entry that were previously possible in the non-fibrotic case (*Figure 5A*). This was possible through the blocking of repeated retrograde excitation, as illustrated earlier in *Figure 3A*. Fibrosis may therefore not always increase arrhythmic risk: failure to sustain early re-excitation in the affected region can be protective. Interestingly, transmural septal fibrosis (*Figure 6A*, right-hand side) increased vulnerability to single re-entries through promoting conduction block at a wider range of S1-S2 coupling intervals, in agreement with *Figure 3B*.

For the alternative AP model (*Figure 6B*), for which arrhythmia was not inducible without diffuse fibrosis in either tissue (*Figure 3C*) or ventricular simulations, sustained rotors became possible for both mid-wall and transmural fibrosis. As in *Figure 3B*, fibrosis had the effect of delaying retrograde propagation in 3D. However, the distribution of fibrosis affected the axis along which conduction was delayed, such that sustained re-entries were enabled for ectopic stimuli at different positions for mid-wall and transmural fibrosis (*Figure 6B*). Sustained rotors were initiated by asymmetry in the retrograde propagation during re-entry caused by the fibrotic microstructure, leading to one side of the figure-of-eight re-entry being blocked by the refractory wavetail, such that a rotor formed around the ischaemic–fibrotic region (*Figure 6C*). As observed before in tissue (*Figure 3C*), this larger configuration of re-entry did not self-terminate, because its long circuit length facilitated an excitable gap that rendered the arrhythmia insensitive to cycle-to-cycle fluctuations in ERP.

## 4. Discussion

Prevention of SCD remains a key challenge in the management of HCM patients, complicated by a limited understanding of SCD causal mechanisms. This study sought to investigate how ionic remodelling and fibrosis can interact with ischaemia in HCM to modulate arrhythmic risk. The principal findings are that (i) during ischaemia, there was a reduction of the ERP and enhanced APD shortening in human HCM cells; (ii) ionic remodelling in HCM enabled re-entry to emerge at a less severe extent of ischaemia; (iii) an increased transmural extent of ischaemia enabled increased arrhythmia sustainability in HCM; and (iv) there were multiple electrophysiology-dependent mechanisms of fibrosis–ischaemia interaction, including the propensity for fibrosis to change figure-of-eight re-entries into more stable patterns of re-entry.

Ischaemic ERP shortening in HCM due to chronic ionic remodelling is not well documented in previous works. However, hyperkalaemic reductions in ERP under conditions of *I*_Kr_ block^43^ (a component of HCM ionic remodelling) have been reported, as opposed to increases in ERP with normal electrophysiology.^44^ In the present study, ischaemic ERP shortening in HCM always occurred concomitantly with enhanced APD shortening (see Supplementary material online, *Figure S10*). Indeed, both enhanced ischaemic ERP^45,46^ and APD^21,45–47^ shortening, and APD dispersion,^48^ have been reported in various hypertrophied animal models. Interestingly, one study instead reported enhanced ischaemic ERP prolongation in hypertrophied rats.^47^ These accentuated responses to ischaemia may in part be explained by faster K^+^ accumulation in regions of hypertrophy,^21^ but the present study identified enhanced ischaemic ERP and APD responses even with the same increase in extracellular K^+^. Although distinct from HCM, LV hypertrophy often manifests prolonged APD at baseline,^7^ and the ischaemic repolarization response is markedly aggravated in these patients,^49^ which may reflect the enhanced cellular response identified in the present study. This aggravated repolarization response could also explain ischaemic T wave pseudonormalization,^50^ whereas the concomitant ischaemic changes to refractoriness may have implications for arrhythmic risk in HCM.

The association of LV hypertrophy with an increased risk of arrhythmias,^7^ and reports that HCM patients with electrocardiographic abnormalities in the S-T complex (evidence of ionic remodelling) have an increased risk of arrhythmias,^51^ may be partially explained by hypertrophy–ischaemia interactions. In one study, canines with LV hypertrophy had a higher rate of SCD during ischaemia compared to their non-hypertrophic counterparts.^52^ Moreover, in rats undergoing ischaemia, ventricular fibrillation susceptibility may be increased in those with LV hypertrophy,^53^ particularly when the ischaemia and hypertrophy coincide.^53^ In the present study, electrophysiological interactions between hypertrophy-associated ionic remodelling and ischaemia increased arrhythmia susceptibility early in ischaemic onset. This is relevant because the severity of demand ischaemia may be self-limiting through dyspnoea and angina in HCM, and ATP levels may be better maintained in demand ischaemia.^54^ Of clinical importance, arrhythmic risk contributions from repolarization impairment in HCM motivate investigation of pharmacologic amelioration of ionic remodelling, through K^+^ channel enhancement or late Na^+^ block. Ranolazine has already shown effect in reducing ectopy burden in HCM,^55^ and this antiarrhythmic effect may extend to the arrhythmic substrate. Aside from the ionic remodelling, it is notable that LV hypertrophy may directly modulate arrhythmic risk through mechanisms akin to the critical mass hypothesis,^56^ and the contribution of this factor relative to the ionic remodelling may be of interest for further study.

Simulations of acute myocardial ischaemia in non-HCM ventricles have demonstrated that multiple re-entrant cycles are more inducible in transmural than subendocardial ischaemia,^12^ a finding reinforced by the present study with HCM ionic remodelling at a lesser extent of ischaemia. The pro-arrhythmic interactions that occur when transmural ischaemia affects HCM-remodelled myocardium may in part explain why adults with both HCM and epicardial coronary artery disease have rates of death that significantly exceed those of patients with just epicardial coronary artery disease.^57^

In previous work that investigated interactions between hypoxia and fibrosis, a deleterious interaction was observed in which hypoxic ERP reductions enabled micro-anatomical re-entries to form in fibrotic myocardium.^38^ However, the present study identified that under conditions of full ischaemia, the net effect on ERP is dependent on the balance of hyperkalaemic and hypoxic severity. When considering hyperkalaemia (and the ionic remodelling in HCM), regional ERP increases are possible. Although ERP increases further precluded micro-anatomical re-entries in the fibrotic simulations, it was identified that in the onset of ischaemia, the increase in ERP heterogeneity instead enabled figure-of-eight re-entries around the affected region. Moreover, in the present study, the impact of fibrosis on arrhythmic risk was critically dependent on its density and fast Na^+^ channel availability, where conduction impairment was initially pro-arrhythmic but became antiarrhythmic as tissue became unexcitable. This effect, identified in previous modelling,^58^ is therefore also relevant in HCM. Additionally, the present study identified that diffuse fibrosis can change re-entry patterns by inducing asymmetric retrograde propagation, breaking the symmetry of figure-of-eight patterns and leading to stable rotor formation.

This study did not investigate how patient-specific geometries and distributions of ischaemia/ionic remodelling, or genotype influence on ionic remodelling, may modulate arrhythmic risk. The region of ischaemia simulated was informed by mid-slice LV perfusion reserve measurements only. Perfusion imaging suggests that there are also patchy distributions of ischaemia in HCM, which would be intricately related to the distribution of ionic remodelling and in many cases not perfectly coinciding. Other types and distributions of fibrosis may also be present in HCM, which may harbour different interactions with ischaemia. The ischaemic BZ was additionally assumed to be identical in width for all components of ischaemia, but differences in the size of the hyperkalaemic and hypoxic borders have been reported. Additionally, we did not investigate possible biphasic patterns of ischaemic K^+^ accumulation in the BZ, where K^+^ concentrations have been reported to decrease in canine^59^ or porcine^60^ models of coronary ligation under longer timescales than the ones considered in this study. The mesh used in all biventricular simulations was derived from an HCM patient with septal hypertrophy; thus, arrhythmic risk modulation by structure (e.g. due to increased muscle mass^61^) was not considered, which may further affect hypertrophy–ischaemia interactions. Conduction velocity in hypertrophied regions was considered as normal based on invasive measurements in the hypertrophied interventricular septum of obstructive HCM patients.^62^ Differences in arrhythmic risk attributable to possible hypertrophy-associated differences in conduction velocity were not considered in the present study. Importantly, despite using a populations of models approach, this study only used the ToR-ORd AP model, and possible dependence of the findings on the choice of baseline AP model was not investigated. This study assumed that ectopic stimuli emerge in the ischaemic BZ as in experimental studies of non-HCM ischaemia; however, there are additional causes of ectopy in HCM that were not considered.^14^ Ectopy and alternans related to Ca^2+^ handling are further expected to be modulated by ischaemic impairment of the SERCA and sarcolemmal Ca^2+^ pumps, despite negligible effects on AP biomarkers (see Supplementary material online, *Figure S11*). Myocardial fibrosis was modelled as unexcitable mesh elements; thus, whether the mechanisms identified in the present study are affected by electrotonic fibroblast–myocyte coupling was not investigated. This study also did not investigate the role that mechanoelectric feedback or the Purkinje system may have on arrhythmogenesis during ischaemia, but the Purkinje system has a minor role in ischaemic re-entries in other computational works.^17^ Further electrophysiological mechanisms are expected to mediate the transition from ischaemia-induced ventricular tachycardia to ventricular fibrillation.

## 5. Conclusions

Combined cardiac electrophysiology simulations and patch-clamp experiments showed that ionic remodelling in HCM causes an accentuated repolarization response and opposite ERP response to acute ischaemia, compared to non-remodelled cells. In tissue and biventricular models, potential deleterious interactions were identified between myocardial ischaemia, repolarization impairment, and diffuse fibrosis. Such mechanisms may be relevant to the occurrence of lethal arrhythmias in patients with mild HCM phenotypic expression, such as in juvenile patients^3^ with no replacement fibrosis^63^ and limited hypertrophic remodelling.^64^

**Translational perspective**

Myocardial ischaemia is a common finding in HCM patients. This study identified an abnormal electrophysiological response of diseased HCM cardiomyocytes to myocardial ischaemia, which manifests as a significantly higher risk substrate for non-sustained ventricular tachycardia, particularly when transmural ischaemia is present in the ventricles. Alteration of ischaemia-induced re-entry patterns by the fibrotic microstructure may induce sustained ventricular tachycardia. Altogether, different interactions between disease factors may increase arrhythmic risk at different stages of the disease: in early disease, ischaemia and repolarization impairment may adversely interact, and then in late disease, there may be further pro-arrhythmic interactions with diffuse fibrosis.

## Supplementary Material

cvae086_Supplementary_Data
