# Supplementary material for: Mechanisms of ischaemia-induced arrhythmias in hypertrophic cardiomyopathy: a large-scale computational study
Source: Cardiovasc Res. 2024 Apr 22;120(8):914–26. doi: 10.1093/cvr/cvae086 (PMC11218689; doi:10.1093/cvr/cvae086)
Supplement: cvae086_Supplementary_Data [file cvae086_supplementary_data.docx]

**Mechanisms of ischaemia-induced arrhythmias in hypertrophic cardiomyopathy: a large-scale computational study**

**Supplementary Material**

James A Coleman^1^, Ruben Doste^1^, Zakariye Ashkir^2^, Raffaele Coppini^3^, Rafael Sachetto^4^, Hugh Watkins^5^, Betty Raman^2,†^, Alfonso Bueno-Orovio^1,†^

^1^Department of Computer Science, University of Oxford, Oxford, United Kingdom

^2^Oxford Centre for Clinical Magnetic Resonance Research (OCMR), Radcliffe Department of Medicine, Division of Cardiovascular Medicine, University of Oxford, Oxford, United Kingdom

^3^Department of NeuroFarBa, University of Florence, Florence, Italy

^4^Department of Computer Science, Federal University of São João del-Rei, Minas Gerais, Brazil

^5^Radcliffe Department of Medicine, Division of Cardiovascular Medicine, University of Oxford, Oxford, United Kingdom

^†^Joint senior authors

Corresponding author: Alfonso Bueno-Orovio alfonso.bueno@cs.ox.ac.uk, Department of Computer Science, University of Oxford, Oxford, United Kingdom

1. **Supplementary methods**

**1.1. Computational model of HCM ionic remodelling**

Ionic remodelling in hypertrophic cardiomyopathy (HCM) cardiomyocytes was modelled through rescaling ToR-ORd action potential (AP) model variables and parameters as detailed in Supplementary Table S1, as in previous works^1,2^ informed by cardiomyocyte currents, protein expression and mRNA data from surgical samples of HCM patients undergoing septal myectomy^3^.

| Variable/Parameter | Symbol | Change |
| --- | --- | --- |
| Cell volume | V | +90% |
| Troponin-Ca^2+^ affinity | K_TRPN_ | -50% |
| Late Na^+^ current | I_NaL_ | +165% |
| Background Na^+^ current | I_Nab_ | +165% |
| Inward K^+^ rectifier current | I_K1_ | -30% |
| Rapidly delayed K^+^ rectifier current | I_Kr_ | -45% |
| Slow delayed K^+^ rectifier current | I_Ks_ | -45% |
| Transient outward K^+^ current | I_to_ | -70% |
| Na^+^/Ca^2+^ exchange current | I_NCX_ | +30% |
| Na^+^/K^+^ pump current | I_NaK_ | -30% |
| Ca^2+^ release current | J_rel_ | -20% |
| Ca^2+^ reuptake current | J_up_ | -25% |
| L-type Ca^2+^ current | I_CaL_ | +40% |
| Fast voltage dependent L-type Ca^2+^ time constant | 𝜏_ff_ | +35% |
| Slow voltage dependent L-type Ca^2+^ time constant | 𝜏_fs_ | +20% |
| Fast Ca^2+^ dependent L-type Ca^2+^ time constant | 𝜏_fcaf_ | +35% |
| Slow Ca^2+^ dependent L-type Ca^2+^ time constant | 𝜏_fcas_ | +20% |
| Voltage shift in jca_∞_ gating | bca_∞_ | +15.0 |

**Supplementary Table S1*.* Model of** **ionic remodelling in HCM.** Up/down-regulation of ion currents was achieved through rescaling of maximum conductances^1,2^.

**1.2. Measuring AP model effective refractory period**

Effective refractory periods (ERPs) were measured in both cellular and cable domains. Cellular ERP measurements were used as estimates of cellular refractoriness to define ranges of S1-S2 coupling intervals for the in-tissue simulations presented in Figure 2. Cable ERP measurements were used as final measurements of ERP, to be reported in the results and figures in the manuscript.

**1.2.1. Cellular effective refractory period**

The ERP of each AP model was measured using an S1-S2 pacing protocol. After AP model stabilisation (100 S1 beats at 1 Hz), a premature stimulus (S2) was applied with the S1-S2 interval increased in 10ms increments. The cellular ERP was defined as the S1-S2 interval for which S2 induced an AP of maximally prolonged upstroke.

**1.2.2. Cable effective refractory period**

The ERP of each AP model was measured using an S1-S2 pacing protocol in a cable of length 5.5cm. After AP model stabilisation (100 S1 beats at 1 Hz in single cell), the S1-S2 protocol consisted of 2 further S1 beats, followed by a premature stimulus (S2) varied in 10ms increments, with S1 and S2 applied at the same end of the cable. The cable ERP was defined as the maximum S1-S2 time interval for which excitation (defined as a membrane potential > -40mV) of the cable centre point did not occur.

**1.3. Experimental patch-clamp methods**

Septal specimens were prepared as we previously described^3^. Briefly, septal samples from HCM patients and non-failing non-hypertrophic aortic stenosis controls who underwent surgical myectomy were rapidly washed in ice-cold cardioplegic solution containing (in mmol/L): KH_2_PO_4_ 50, MgSO_4_ 8, HEPES 10, adenosine 5, glucose 140, mannitol 100, taurine 10 (pH 7.4 with KOH). Samples were then minced to small pieces (~1mm^3^) and subjected to enzymatic and mechanical dissociation to obtain viable single myocytes, as described before^4^. In brief, small tissue chunks were transferred into a scraping device, while the bathing solution was changed to Ca^2+^-free dissociation buffer containing (in mM): NaCl 113, KCl 4.7, KH_2_PO_4_ 0.6, Na_2_HPO_4_ 0.6, MgSO_4_-7H_2_O 1.2, NaHCO_3_ 12, KHCO_3_ 10, HEPES 10, taurine 20, Na pyruvate 4, glucose 10, BDM 10 (pH 7.3 with NaOH) and heated to 37 ºC. Collagenase Type V and Protease Type XXIV (Sigma) were subsequently added and tissue chunks digested for a total 2 hours’ time. During the digestion, the buffer containing dissociated myocytes was collected every 15 minutes from the scraping device and diluted with KB solution at room temperature. KB solution contained (in mM): KCl 20, KH2PO4 10, glucose 25, mannitol 5, L-glutamic acid monopotassium salt 70, β-hydroxybutyric acid 10, EGTA 10 and 2mg/mL albumin (pH 7.2 with KOH). The myocytes were left to settle and then resuspended in Ca^2+^-free Tyrode solution containing (in mM): 132 NaCl, 5 KCl, 1.2 MgCl_2_ 10 HEPES, and 11 glucose (pH 7.35 NaOH), supplemented with bovine serum albumin (1mg/ml). CaCl_2_ was added stepwise up to 0.6 mM. Cells were stored in this solution and used within 3 hours. Cells were washed and transferred to a temperature-controlled recording chamber (experimental temperature= 35±0.5ºC), mounted on the stage of an inverted microscope. Current-clamp experiments were conducted as previously^3^. Action potentials (APs) were measured simultaneously using the perforated-patch configuration (amphotericin-B method). Specifically, for AP recordings, the pipette solution contained (in mM) 115 K methanesulfonate, 25 KCl, 10 HEPES, 3MgCl_2_ and cells were superfused with Tyrode buffer (see above) containing 1.8mM CaCl_2_. APs were elicited with short depolarizing stimuli (<3ms) at different frequency of stimulation (0.2Hz and 0.5Hz, 1 minute at each frequency). Extracellular [K] was then raised from 5mM up to 9mM and AP recordings were repeated using the same stimulation protocol.

**1.4. Clinical perfusion imaging data**

| **Demographic / Clinical Data** | |
| --- | --- |
| Age (years) | 49 ± 14 |
| Male sex | 23 (82%) |
| Weight (kg) | 82 ± 5 |
| Height (cm) | 175 ± 8 |
| Body mass index (kg/m^2^) | 27 ± 4 |
| LV outflow tract obstruction | 9 (32%) |
| Ventricular tachycardia | 5 (18%) |
| **CMR** | |
| LVEDV (ml) | 166 ± 32 |
| LVESV (ml) | 49 ± 16 |
| Stroke volume (ml) | 117 ± 24 |
| Ejection fraction (%) | 71 ± 7 |
| Max wall thickness (mm) | 20 ± 4 |
| **Segmental MPRI (Mid LV)** | |
| Anterior | 1.3 ± 0.4 |
| Anteroseptal | 1.1 ± 0.4 |
| Inferoseptal | 1.2 ± 0.4 |
| Inferior | 1.3 ± 0.5 |
| Inferolateral | 1.5 ± 0.5 |
| Anterolateral | 1.3 ± 0.4 |

**Supplementary Table S2*.* Characteristics and demographics of HCM patients (n = 28) that underwent perfusion imaging.** LV: left ventricular; LVEDV: LV end-diastolic volume; LVESV: LV end-systolic volume.

1. **Supplementary Computational Results**

**2.1. Effects of acidotic impairment of Na^+^ and L-type Ca^2+^ channels**


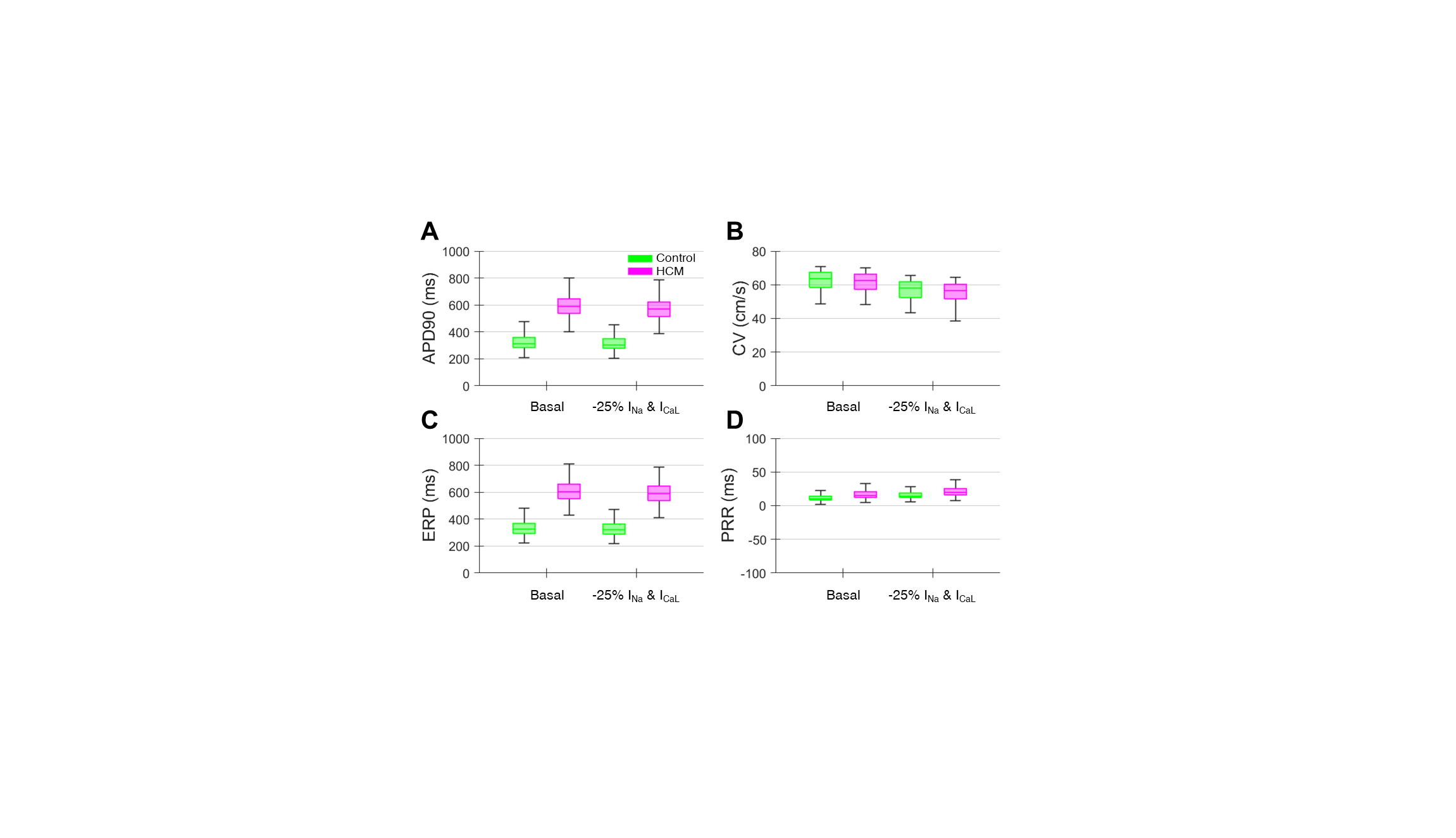
Acidosis alone had small effects on ERP in both control (330±60 vs. 330±60 ms; acidosis vs. baseline) and HCM (590±80 vs. 600±80 ms) AP models (Supplementary Figure S1). The main consequence of acidosis was reduced conduction velocity in control (57±6 vs. 63±6 cm/s; acidosis vs. baseline) and HCM (56±5 vs. 62±6 cm/s). Therefore, no substantial differences in the response to acidosis between control and HCM AP models were observed.

**Supplementary Figure S1*.* Effects of acidotic impairment of Na^+^ and L-type Ca^2+^ channels on AP biomarkers in control and HCM populations of AP models.** (A) Action potential durations, (B) conduction velocities, (C) effective refractory periods and (D) postrepolarisation refractoriness for control and HCM populations of models subjected to basal conditions and acidosis (-25% I_Na_ & I_CaL_).

**2.2. Ionic mechanisms underlying abnormal response of HCM AP models to ischaemia**

The elevation of resting membrane potential (RMP) associated with hyperkalaemia in myocardial ischaemia significantly modulates the recovery of inactivation of the late sodium current (I_NaL_). This is illustrated in Supplementary Figure S2 by the maximal (steady state) magnitudes of I_NaL_ inactivation gating. Such a reduction in maximal open channel probability directly translates into decreased I_NaL_ magnitude (Supplementary Figure S2A & B), therefore contributing to AP shortening. As ionic remodelling in HCM is characterised by a stark upregulation of I_NaL_ channels^3^, I_NaL_ impairment by hyperkalaemia is thus magnified in HCM. This large difference in sustained inward current between basal and ischaemic conditions leads to enhanced AP shortening of HCM cardiomyocytes when undergoing ischaemia. Similarly, the rapid delayed rectifier potassium current (I_Kr_) is significantly downregulated by ionic remodelling in HCM^3^. Systematically removing this component of HCM ionic remodelling (i.e., increasing I_Kr_) therefore increases the magnitude of total repolarisation current, shortening AP duration and bringing it closer to that of control cardiomyocytes (Manuscript Figure 1E). Changes in ERP associated with I_Kr_ downregulation under ischaemia were however due to interactions between I_Kr_ and I_K1_, where combined prolongation of the plateau phase due to HCM remodelling in I_Kr_ and the effect of [K^+^]_o_ on I_K1_ voltage-dependence led to an enhanced hyperkalaemic repolarisation response, in agreement with experimental findings in rabbit ventricular cardiomyocytes^5^.

**
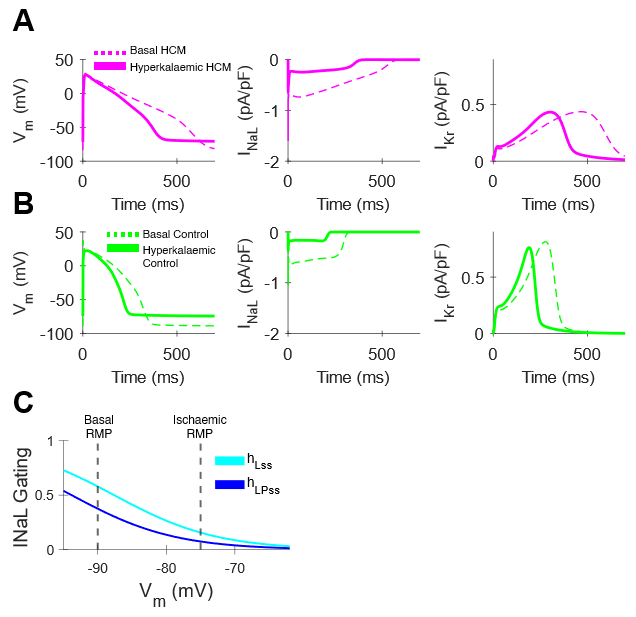
**

**Supplementary Figure S2**. **Remodelling of late sodium (I_NaL_) and rapidly delayed rectifier (I_Kr_) channels underlies abnormal response of HCM cardiomyocytes to ischaemia.** Representative AP traces (left), I_NaL_ (middle) and I_Kr_ (right) under basal (dashed line) and hyperkalaemic (K_o_=9mM) (solid line) conditions, for (A) HCM-remodelled cardiomyocytes, and (B) control cardiomyocytes. (C) Non-phosphorylated (h_Lss_) and phosphorylated (h_LPss_) inactivation gating steady states of I_NaL_, where there is significant impairment during ischaemic RMP elevation compared with basal RMP (dashed, vertical lines). V_m_: membrane potential.

**2.3. Effects of ischaemia on Ca^2+^ handling**

The impact of acute myocardial ischaemia on Ca^2+^ handling in the populations of control and HCM AP models was also investigated (Supplementary Figure S3). At baseline, HCM AP models had longer Ca^2+^ transient durations than controls (530±50 vs. 390±50 ms; HCM vs control), elevated diastolic Ca^2+^ (82±7 vs. 77±5 nM) and lower Ca^2+^ transient amplitudes (340±90 vs. 380±80 nM), as reported experimentally^3^.

Ca^2+^ transient amplitudes were reduced similarly in control and HCM populations during hyperkalaemia (-60±40 vs. -80±60 nM), hypoxia (-120±30 vs. -90±40 nM), acidosis (-150±30 vs. -130±40 nM) and ischaemia (-240±50 vs. -220±80 nM).

Small changes in Ca^2+^ transient durations were observed in control and HCM populations during hyperkalaemia (+8±10 vs. +7±20 ms) and hypoxia (+9±10 vs. 0±20 ms). During acidosis, Ca^2+^ transient durations were increased similarly in control and HCM populations (+40±5 vs. +40±20 ms), and this was also observed during ischaemia (+50±20 vs. +40±40 ms).

**
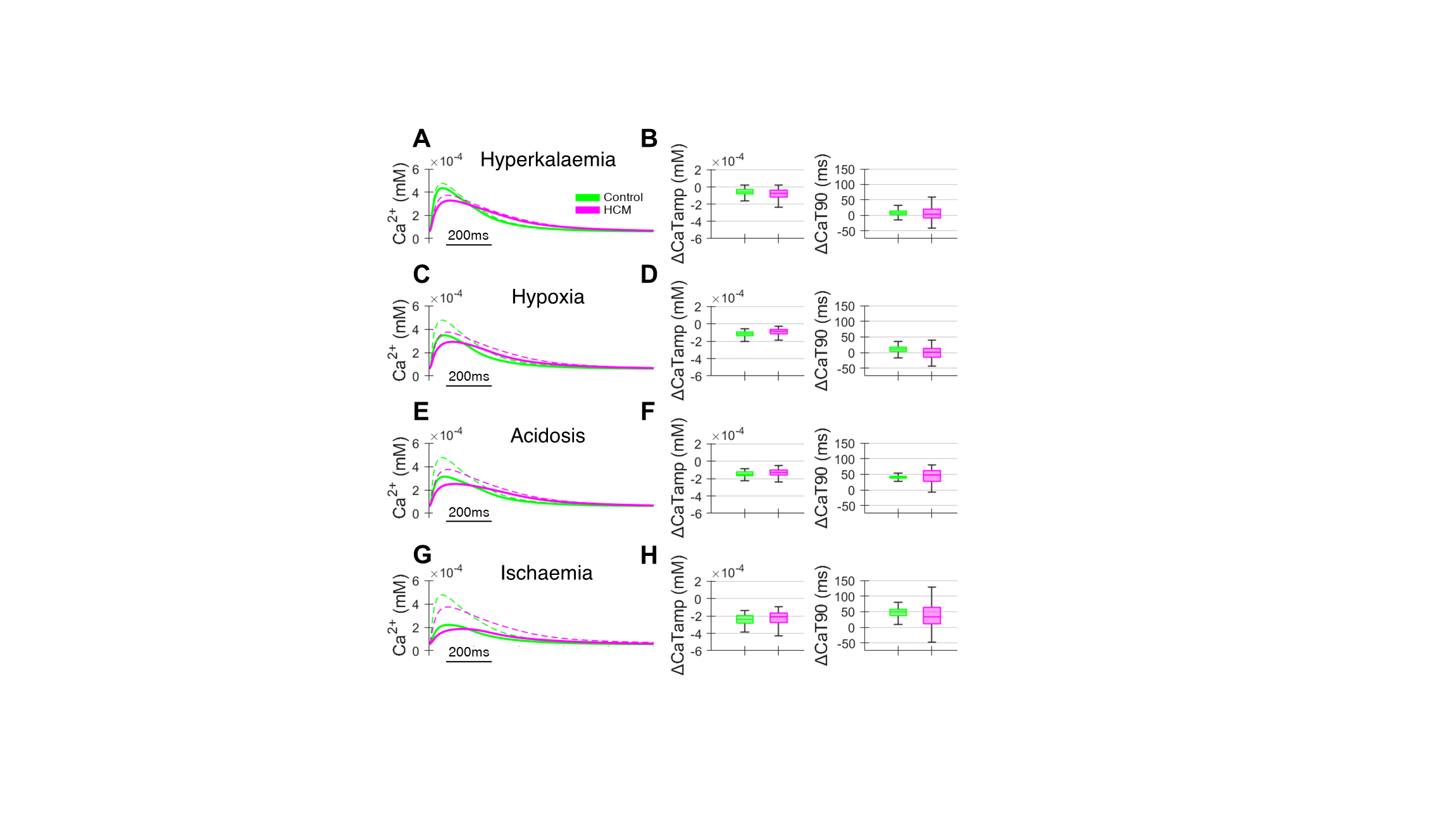
**

**Supplementary Figure S3*.* Effects of ischaemia on Ca^2+^ handling in control and HCM populations of AP models.** (A, C, E, G) Representative Ca^2+^ transients under basal conditions (dashed) compared to during (A) hyperkalaemia (K_o_ = 9mM), (C) hypoxia (f_KATP_ = 0.09), (E) acidosis (-25% I_Na_ & I_CaL_) and (G) ischaemia (K_o_ = 8mM, f_KATP_ = 0.06, -25% I_Na_ & I_CaL_), (solid). (B, D, F, H) Changes in Ca^2+^ transient amplitudes (ΔCaTamp) (left) and changes in Ca^2+^ transient durations measured at 90% decay (ΔCaT90) (right) undergone for each respective condition.

In summary, myocardial ischaemia led to similar prolongation of Ca^2+^ transients and similar impairment of Ca^2+^ transient amplitudes in control and HCM AP models. The Ca^2+^ durations which are prolonged in HCM models under basal conditions remained significantly prolonged during ischaemia compared to controls ((+570±30)ms vs. (+440±50)ms), which contrasts with the changes observed in APD in HCM models.

**2.4. Sensitivity of AP biomarkers to ischaemic effects on I_NaK_, I_NaL_ and I_NCX_**

Although hyperkalaemia, hypoxia and acidosis are the main causes of electrical changes during acute myocardial ischaemia^6^, there are various formulations of hypoxic and acidotic effects in computational cardiac electrophysiology models. In addition to hypoxic activation of I_KATP_ and the acidotic effects on I_Na_ and I_CaL_ modelled in the present study and in previous works^7–9^, more detailed models of hypoxia and acidosis include changes in I_NaK_ and I_NCX_^10^. Furthermore, the accumulation of the ischaemic metabolite lysphosphatidylcholine and its upregulation of I_NaL_^10^ may be of relevance to the present study, in which ionic remodelling in I_NaL_ is a key component of HCM cells. The sensitivity of our results to these additional ischaemic effects were analysed by repeating cellular and in-tissue simulations with changes in I_NaK_, I_NCX_ and I_NaL_ extracted directly from a more detailed computational model of ischaemia^11^.

Supplementary Figure S4 shows how AP biomarkers were affected by these additional ischaemia components. Compared with the original model of ischaemia, the inclusion of moderate changes in I_NaK_/I_NCX_/I_NaL_ led to longer APDs in control cells (190±40 vs. 180±50 ms), an effect which was slightly more pronounced in HCM cells (330±50 vs. 310±50 ms) due to basal I_NaL_ upregulation in HCM (Supplementary Figure S4A). There were also marginal reductions in CV in control myocardium (44±6 vs. 45±6 cm/s) due to the less negative RMP caused by impairment of I_NaK_, which was slightly more pronounced in HCM cells (37±7 vs. 40±6 cm/s) (Supplementary Figure S4B). Differences in ERPs (Supplementary Figure S4C) were largely secondary to differences in APDs, where moderate changes in I_NaK_/I_NCX_/I_NaL_ led to longer ERPs in control cells (310±60 vs. 290±50 ms), an effect which was again more pronounced in HCM cells (490±90 vs. 460±80 ms). PRRs (Supplementary Figure S4D) were largely unchanged in control cells (120±50 vs. 120±70 ms) but were slightly increased in HCM cells (160±70 vs. 150±60 ms). When compared with non-ischaemic cells, all models of ischaemia caused similar changes to AP biomarkers in both control and HCM.

**
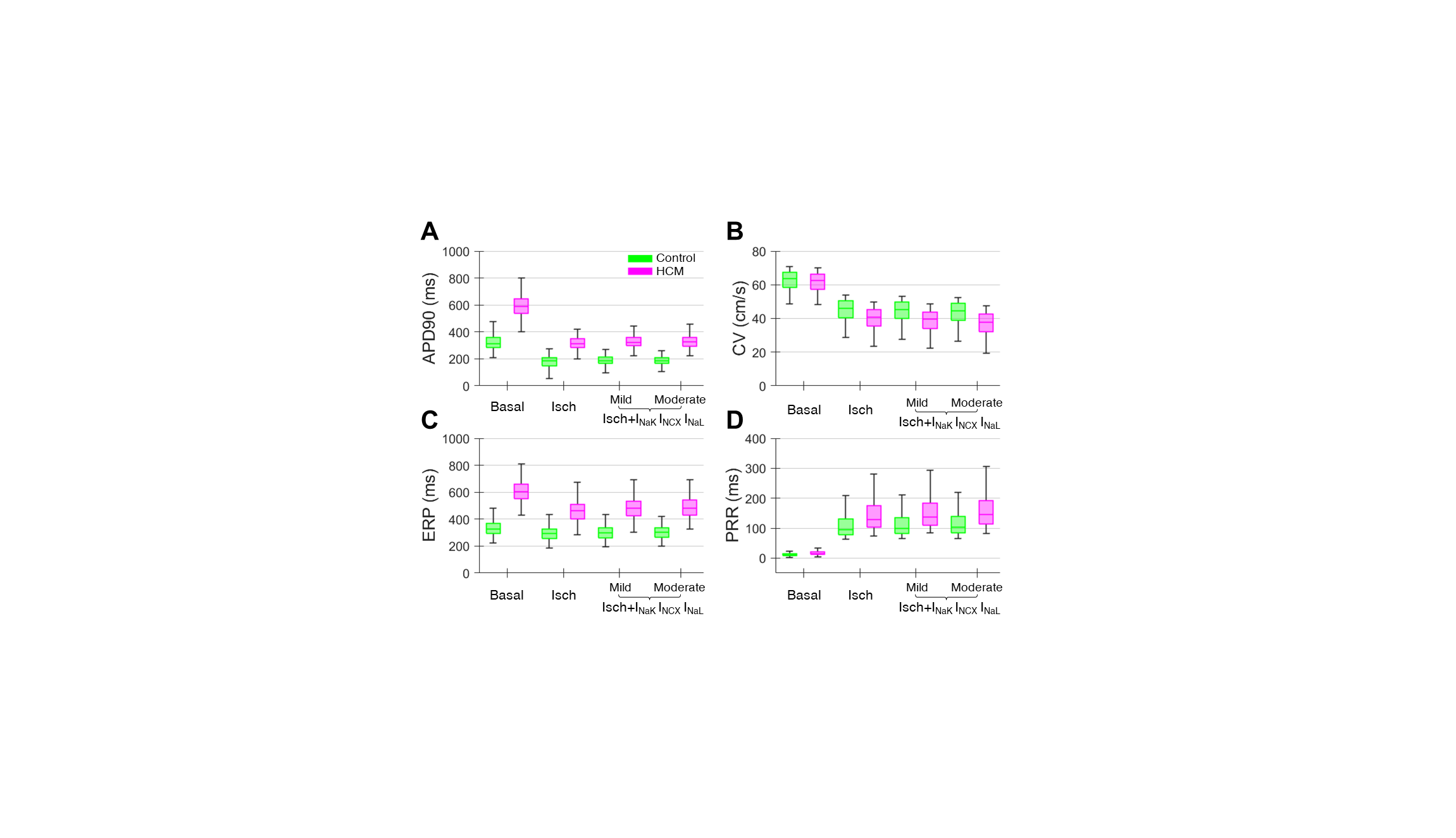
**

**Supplementary Figure S4*.* Effects on AP biomarkers of extending the model of ischaemia to include changes in I_NaK_, I_NCX_ and I_NaL_.** (A) Action potential durations, (B) conduction velocities, (C) effective refractory periods and (D) postrepolarisation refractoriness for control and HCM populations of models subjected to basal conditions, the original ischaemia conditions (K_o_=8mM, f_KATP_=0.06, -25% I_Na_ & I_CaL_), and ischaemia with mild/moderate effects on I_NaK_ I_NCX_ I_NaL_ (K_o_=8mM, f_KATP_=0.06, -25% I_Na_ & I_CaL_, -20%/-40% I_NaK_ & I_NCX_, +40%/+70% I_NaL_).

**2.5. Increased propensity to conduction failure in ischaemic HCM tissue**

Despite significant gradients of refractoriness and reductions in conduction velocity at a severe extent of hyperkalaemia ([K*^+^*]_o_=9mM), re-entry in tissue models of HCM was not inducible. In ectopic S1-S2 protocols, retrograde propagation of S2 typically failed in HCM due to a lack of sodium current availability (Supplementary Figure S5). Under consideration of HCM ionic remodelling, the inclusion of Na^+^/K^+^ pump impairment^1^ leads to further enhanced RMP elevation, which impairs sodium gating, leading to a reduced peak sodium current.


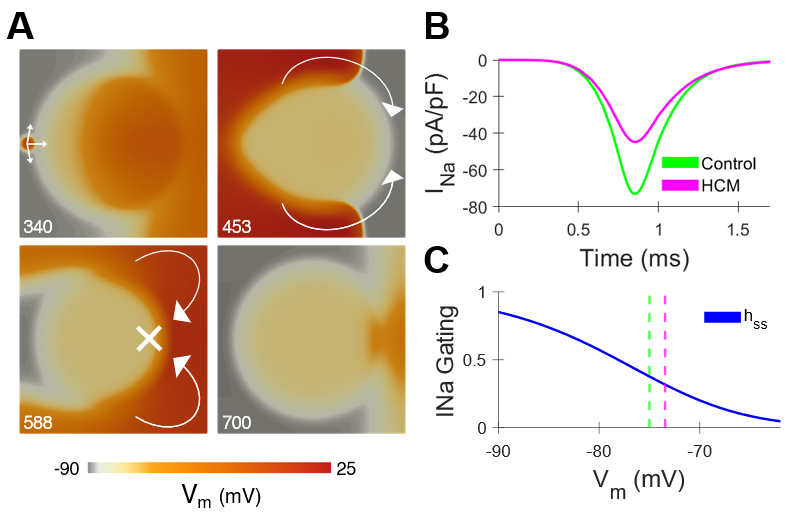


**Supplementary Figure S5*.* Mechanisms of retrograde propagation failure in HCM tissue at high hyperkalaemic extents.** (A) Retrograde propagation failure occurred in most HCM tissue models at a high extent of hyperkalaemia ([K*^+^*]_o_=9mM), precluding re-entry. The time elapsed (ms) following S1 is denoted in each frame. (B) Sodium current availability at K_o_ = 9mM is significantly impaired, especially so in HCM models. (C) Sodium current impairment is contributed to by enhanced RMP elevation in HCM models (magenta, dashed) compared to control models (green, dashed). HCM models had impairment of Na^+^/K^+^ pump function, further elevating RMP and decreasing steady state inactivation gating of I_Na_.

**2.6. Competing effects of increasing severity of ionic remodelling on arrhythmic risk**

As in previous work^1^, our modelling of HCM ionic remodelling was informed by cardiomyocyte current measurements, protein expression, and mRNA data from surgical samples of HCM patients undergoing septal myectomy^3^. However, patients undergoing septal myectomy may represent a narrow window of advanced disease progression. Evidence from murine models suggests that ionic remodelling in HCM is instead progressive^12^. To further investigate how arrhythmic risk might be sensitive to the degree of ionic remodelling in HCM, arrhythmic risk studies were performed in ischaemic HCM tissue for a range of regional K^+^ channel blocks in [50%, 60%, 70%, 75%, 80%] to characterise different extents of AP prolongation as a primary hallmark of the HCM cellular phenotype. This involved applying ectopic S1-S2 stimulus protocols to regionally ischaemic (K_o_ = 7mM, f_KATP_ = 0.06) tissue, across 50 AP models, for variable degrees of ionic remodelling.

Supplementary Figure S6 shows how arrhythmic risk varied across the considered 50 AP models as the APD of the ischaemic region was increased. Arrhythmic risk (both single and multiple re-entries) did not monotonically increase with increasing APD, illustrating competing mechanisms of ionic remodelling in HCM on arrhythmic risk. As the APD of the core ischaemic HCM zone increased from (260±30)ms to (340±40)ms, arrhythmic risk was increased because of the increasing heterogeneity of refractoriness, which enabled conduction block at a wider range of S1-S2 coupling intervals. However, as the APD of the ischaemic HCM region increased beyond (340±40)ms, arrhythmic risk decreased. This occurred because, for the ischaemic region to have recovered excitability after the S1 stimulus, the S2 ectopic stimulus must be applied at increasingly late coupling intervals, otherwise retrograde propagation in the HCM ischaemic region becomes unattainable due to refractoriness in this region. However, as the S1-S2 coupling interval is increased with increasing APD, the conduction velocity for the S2 stimulus also increases as sodium channels increasingly recover. The faster propagation of the S2 stimulus began to impair many re-entries, such that arrhythmic risk decreased.

An additional mechanism further underlies the relative infrequency of multiple cycles of re-entry when compared to single re-entries. Whereas for achieving single re-entries the S2 ectopic stimulus can be applied at a late coupling interval so that the ischaemic region has almost fully recovered excitability (as discussed above), a further condition sensitive to the ERP of the ischaemic HCM zone holds for additional cycles of re-entry: the ERP must be low enough ($ERP<\frac{l}{v}$) to allow re-excitation for a given circuit length $l$ at conduction velocity $v$. This underlies the smaller propensity of multiple cycles of re-entry and their associated smaller mean vulnerable window compared to single re-entries, as depicted in Supplementary Figure S3.

Altogether, these findings indicate that ischaemia-induced arrhythmic risk in HCM may progressively increase in the early stages of HCM as the disease-associated ionic remodelling develops, but this may be partially subverted at later stages of the disease where more sizeable regional increases in APD/ERP counterbalance re-entries.


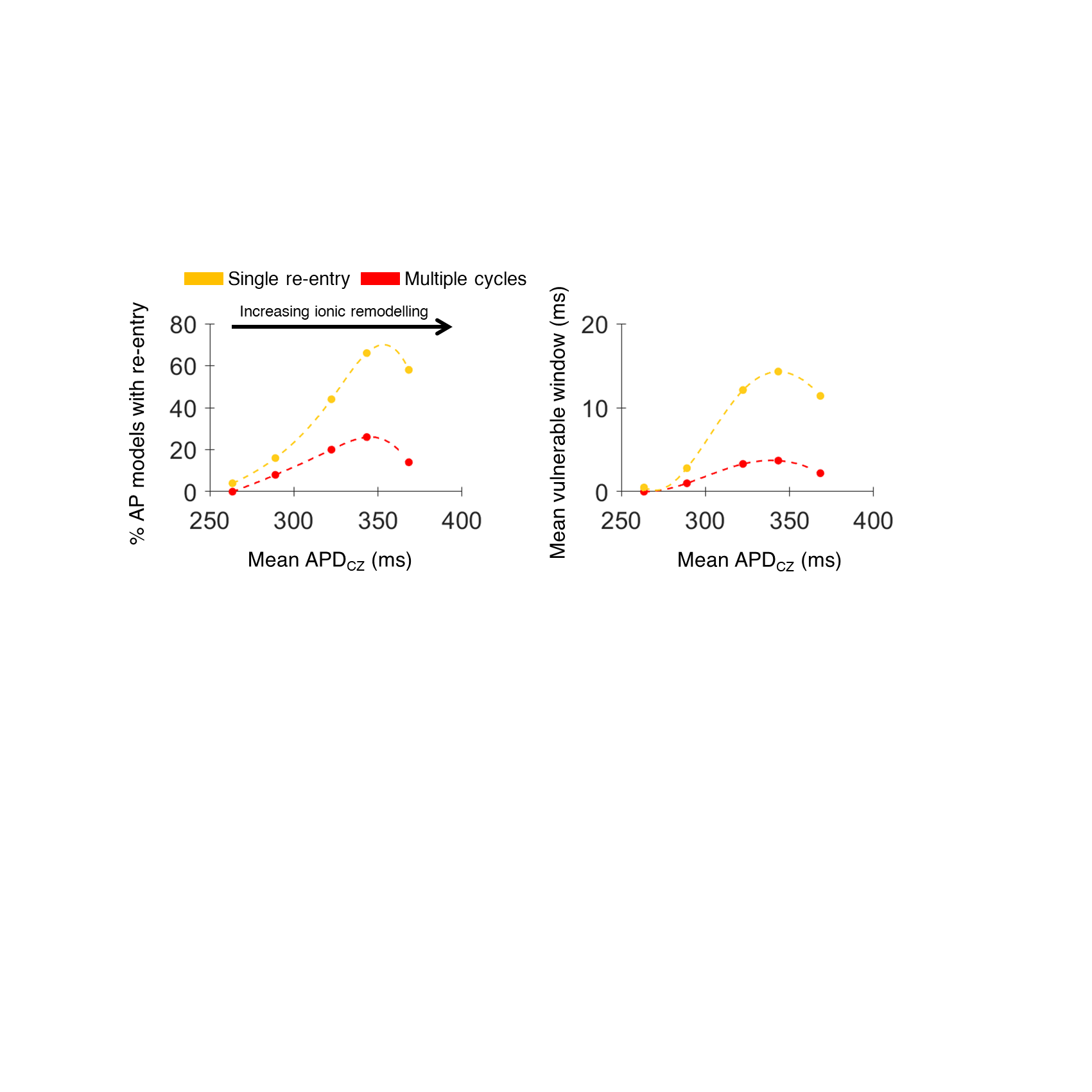


**Supplementary Figure S6. Modulation of ischaemic arrhythmic risk by extent of HCM ionic remodelling.** Summary statistics of arrhythmic risk over the population of 50 AP models, subjected to regional ischaemia with K_o_ = 7mM and f_KATP_ = 0.06, for I_Kr_, I_K1_ and I_Ks_ block in [50%, 60%, 70%, 75%, 80%]. Increasing block of K^+^ currents led to increased arrhythmic risk up to a peak in APD, beyond which arrhythmic risk fell.

**2.7. Sensitivity of in-tissue arrhythmic risk to ischaemic effects on I_NaK_, I_NaL_ and I_NCX_**

Supplementary Figure S7 shows how arrhythmic risk as measured in tissue was affected by extending the model of ischaemia to include effects on I_NaK_, I_NCX_ and I_NaL_. When compared with the original ischaemia conditions (Supplementary Figure S7A), the more detailed model of ischaemia (Supplementary Figure S7B) supported similar patterns of arrhythmic risk across the hyperkalaemia/hypoxia parameter space, with re-entries again occurring at a lesser hyperkalaemic extent in HCM models than in controls. As before with the AP biomarkers, the sensitivity of arrhythmic risk to ischaemic changes in I_NaK_, I_NCX_ and I_NaL_ was greater in HCM models, due to basal upregulation of I_NaL_ and reduced conduction reserve in HCM.

**Supplementary Figure S7*.* Effects on in-tissue arrhythmic risk of extending the model of ischaemia to include changes in I_NaK_, I_NCX_ and I_NaL_.
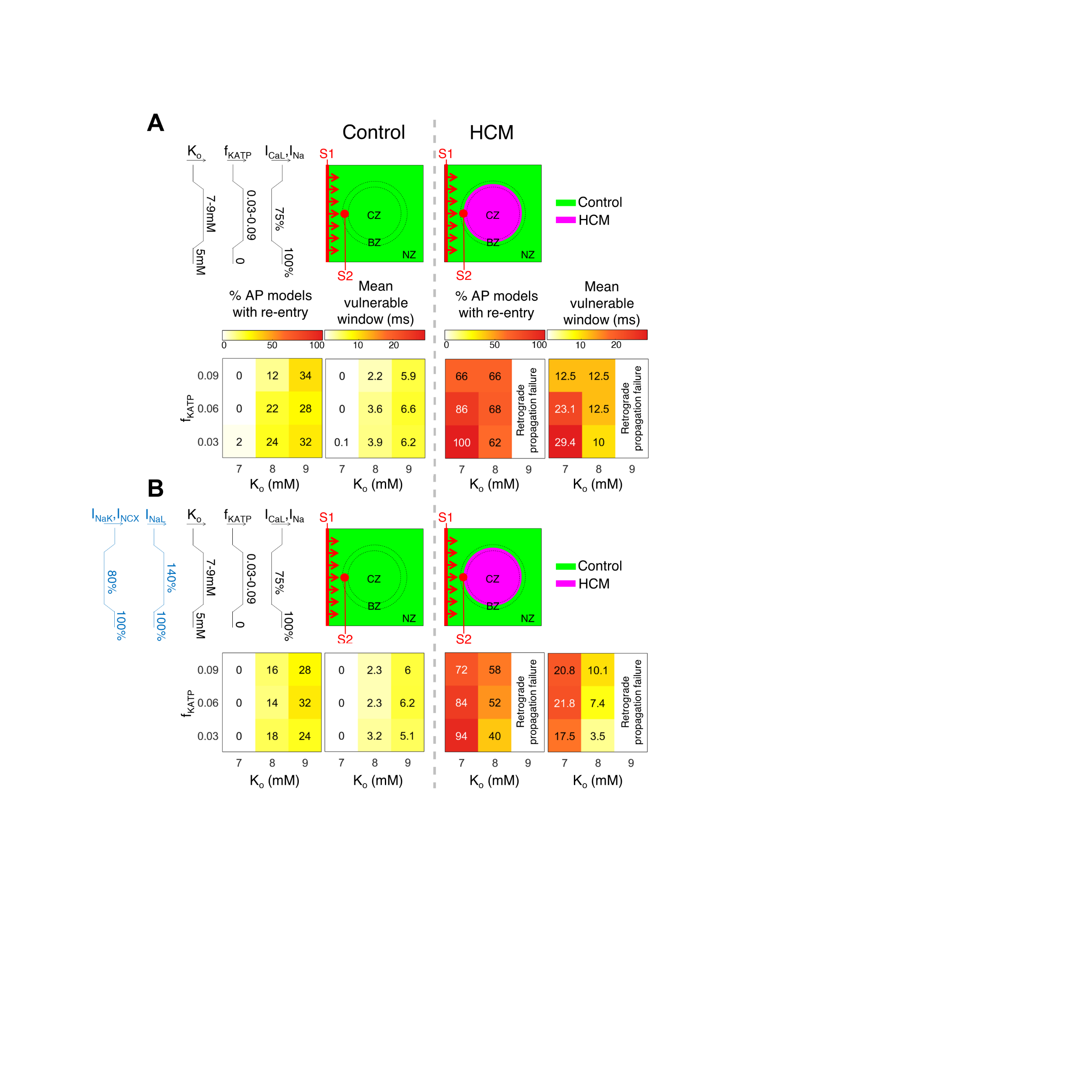
**Summary statistics of arrhythmic risk over the hyperkalaemia/hypoxia parameter space for the 50 AP models in tissue, as characterised using S1-S2 pacing protocols, for (A) the original ischaemia conditions (K_o_=7-9mM, f_KATP_=0.03-0.09, -25% I_Na_ & I_CaL_) and (B) ischaemia with mild effects on I_NaK_ I_NCX_ I_NaL_ (K_o_=7-9mM, f_KATP_=0.03-0.09, -25% I_Na_ & I_CaL_, -20% I_NaK_ & I_NCX_, +40% I_NaL_).

**2.8. Sensitivity of in-tissue arrhythmic risk to hypoxic BZ width**


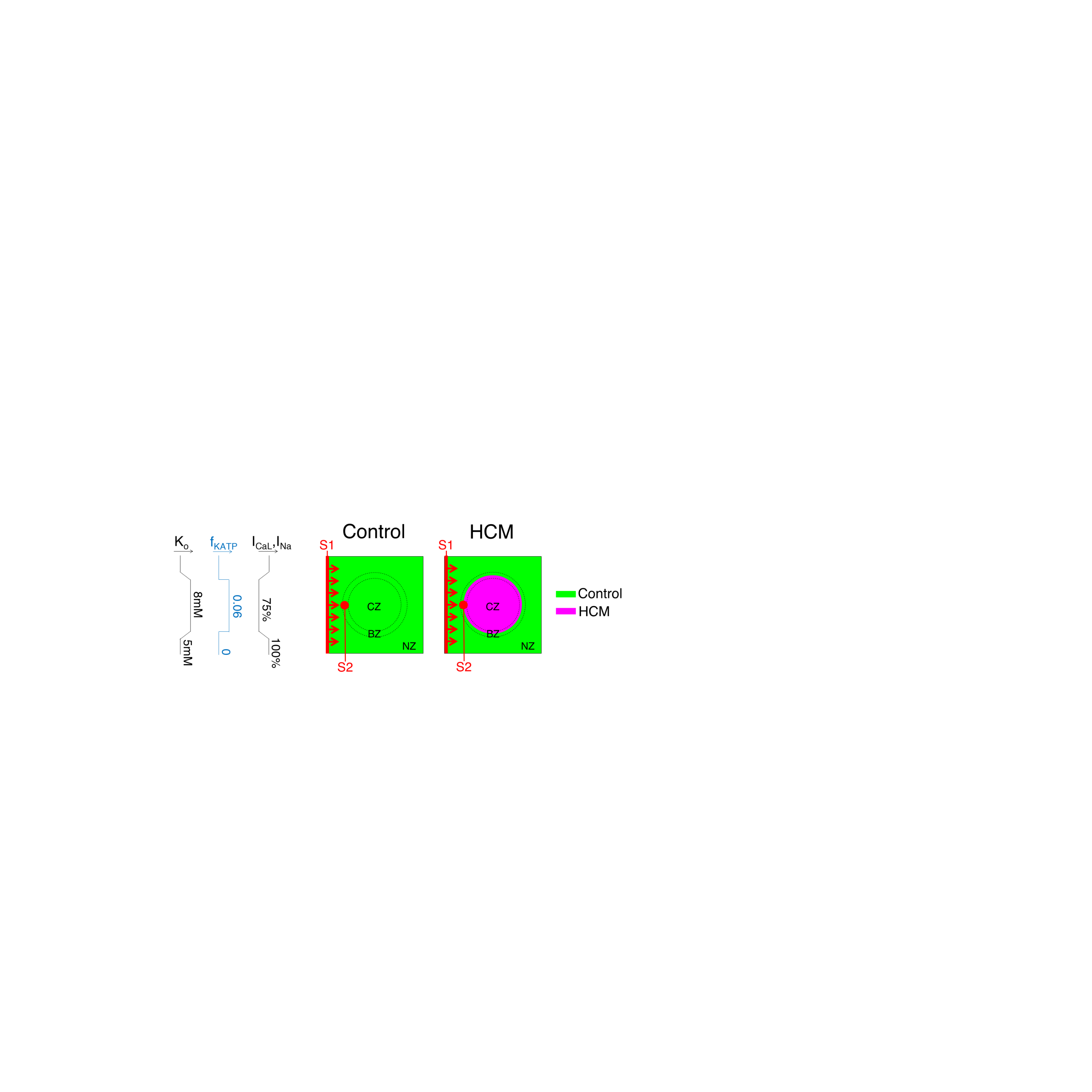
The in-tissue models of ischaemia assumed that the lateral BZ width of hyperkalaemia, hypoxia and acidosis were equal^13^. Previous works have used different lateral BZ widths for the components of ischaemia^10^, to include steeper gradients of ATP than K^+^ in the lateral BZ. To analyse whether the choice of BZ affected the in-tissue results, in-tissue simulations at K_o_=8mM, f_KATP_=0.06 were repeated for a sharp transition in K-ATP channel activation (no hypoxic BZ) as shown in Supplementary Figure S8.

**Supplementary Figure S8. Tissue domains used to test the sensitivity of arrhythmic risk to the hypoxic lateral BZ width.**

Compared to simulations with a BZ in hypoxia (Supplementary Figure S7A), the mean vulnerable window width and the percentage of AP models with re-entry was similar in simulations without a hypoxic BZ (2.7ms, 20% vs. 3.6ms, 22% with hypoxic BZ, in controls). This also applied to HCM tissue models (14.0ms, 74% vs. 12.5ms, 68% with hypoxic BZ).

**2.9. Sensitivity of in-tissue arrhythmic risk to spatial resolution**

The effects of varying spatial resolution on conduction is shown in Supplementary Figure S9. Longitudinal and transverse/transmural conductivities were tuned such that CVs in cable simulations agreed with experimental measurements made in humans^14^ for computationally feasible spatial resolutions (Supplementary Figure S9A & S9B). Ischaemic conduction velocities were further measured in 1D cable simulations for [K^+^]_o_ = [7, 8, 9]mM, corresponding to v_longitudinal_ in [58, 50, 40]cm/s, such that mild hyperkalaemic extent (7mM) was consistent with human measurements of longitudinal conduction velocity after 3min of ischaemia^14^. Tissue simulations used isotropic conductivities (σ_x_ = σ_y_ = σ_z_ = 2.52mS/cm) and a spatial discretisation of 250μm. Biventricular simulations used anisotropic conductivities relative to fibre orientation (σ_longitudinal_ = 2.52mS/cm and σ_transverse_ = σ_transmural_ = 1.57mS/cm) and a spatial discretisation of 300μm.

To analyse how spatial resolution affected the in-tissue results, in-tissue simulations in ischaemic control models (n=50) at K_o_=8mM, f_KATP_=0.06 were compared between dx=[200, 250, 500]μm. The corresponding mean vulnerable window widths were [3.1, 3.6, 2.6]ms, and corresponding the percentages of AP models with re-entry were [20, 22, 18]%, showing modest sensitivity to dx at the AP population level. In individual tissue models, despite the occurrence of re-entry being similar, the patterns of re-entry were not identical, reflecting greater CV at dx=200μm (Supplementary Figure S9C) than at dx=500μm (Supplementary Figure S9D).

**
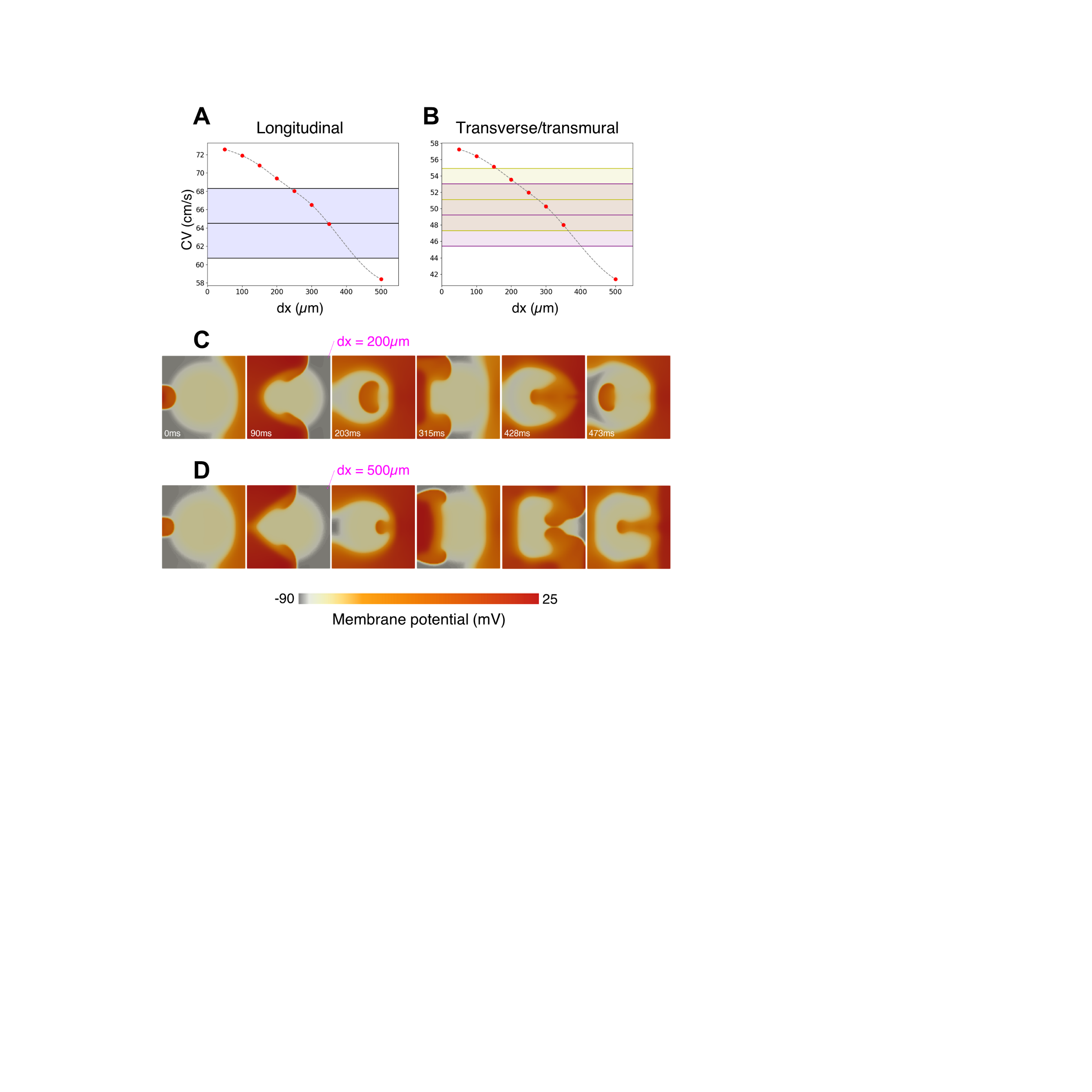
Supplementary Figure S9. Effects of spatial resolution on conduction.** (A, B) conduction velocity (CV) vs. spatial resolution (dx) as measured in cable under control conditions in the baseline ToR-ORd AP model, in the (A) longitudinal (σ_longitudinal_ = 0.000252) and (B) transverse/transmural (σ_transverse_ = σ_transmural_ = 0.000157) directions. Highlighted regions in CV correspond to human experimental measurements of CV (mean ± SEM) along the longitudinal (blue), transverse (purple) and transmural (yellow) directions^14^. (C, D) Representative re-entries in ischaemic control tissue using spatial resolutions (C) dx = 200μm and (D) dx = 500μm. Time elapsed following S2 (ms) is denoted in each frame.


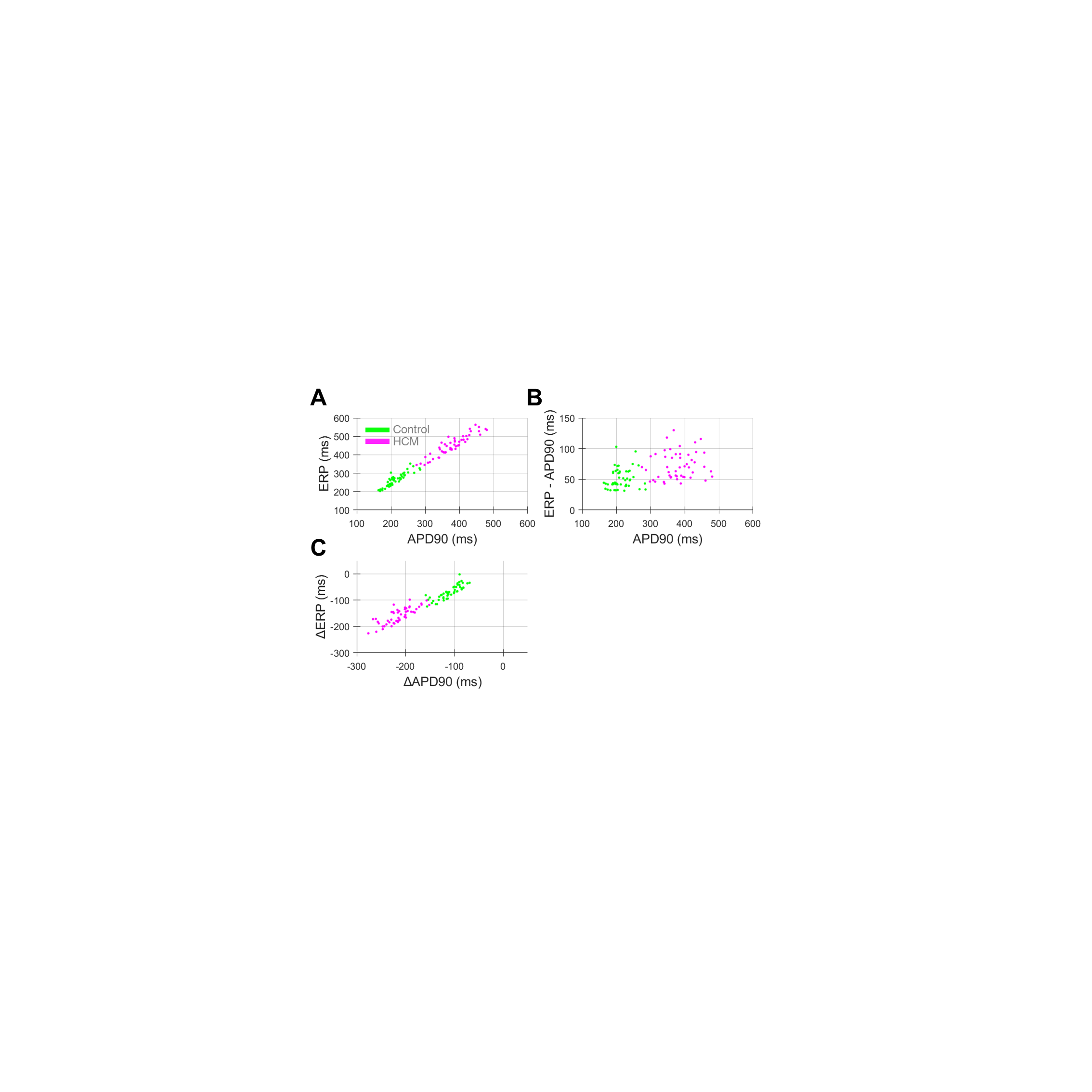
**2.10. Repolarisation and refractoriness during ischaemia**

**Supplementary Figure S10**. **Refractoriness and repolarisation under ischaemic conditions (K_o_=7mM, f_KATP_=0.06, -25% I_Na_ & I_CaL_, -20% I_NaK_ & I_NCX_, +40% I_NaL_), as measured in n=50 control and HCM cable simulations.** (A) Effective refractory period (ERP) and (B) post-repolarisation refractoriness, vs. action potential duration (APD90). (C) The difference in ERP during ischaemia with respect to basal conditions (ΔERP) vs. the difference in APD90 during ischaemia with respect to basal conditions (ΔAPD90).


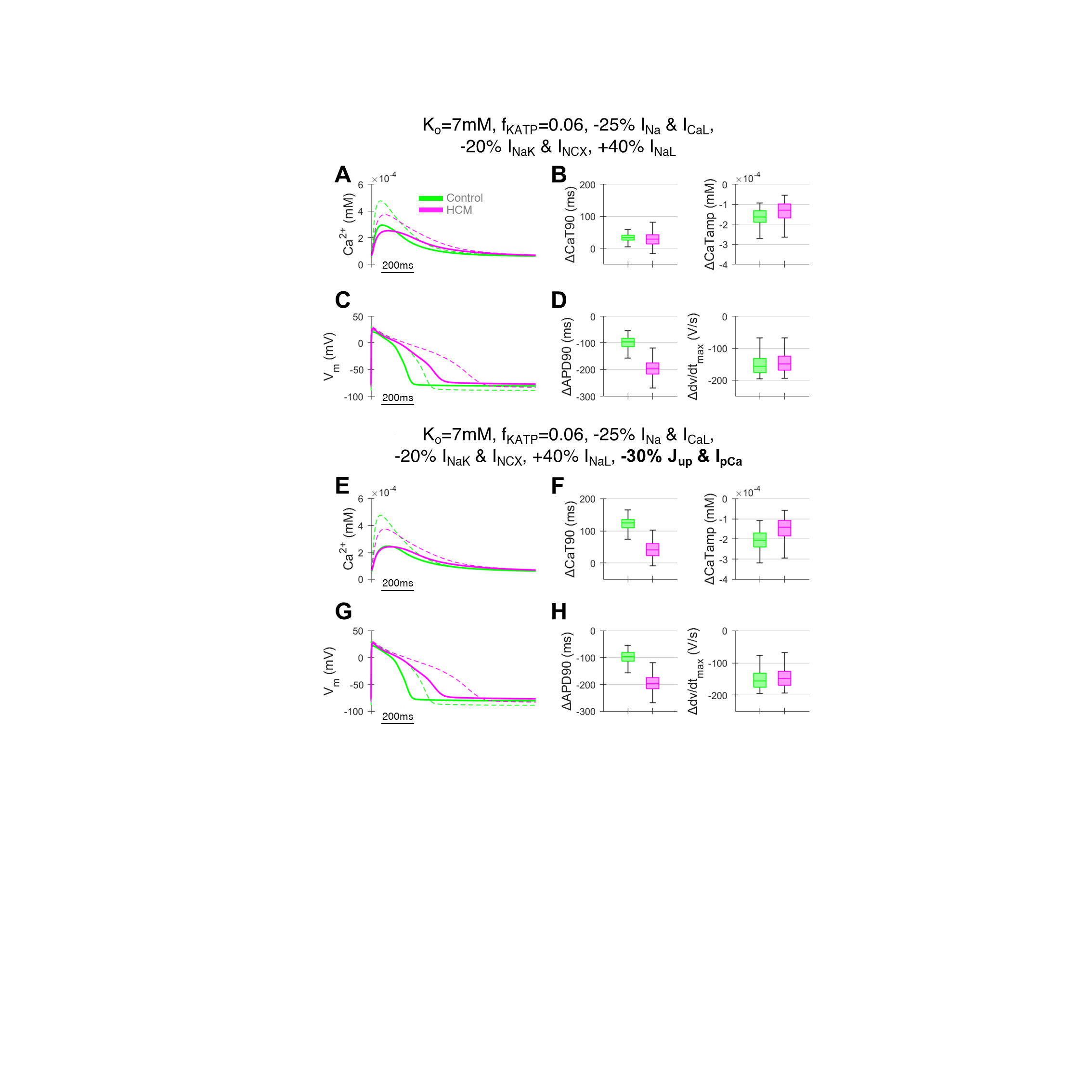
**2.11. Sensitivity of AP biomarkers to further ischaemic effects on J_up_ and I_pCa_**

**Supplementary Figure S11*.* Effects on AP and Ca^2+^ transient biomarkers of further extending the model of ischaemia to include impairment of J_up_ and I_pCa_.** (A, E) Representative Ca^2+^ transients and (C, G) representative AP traces, under basal conditions (dashed) compared to (A, C) ischaemia without impairment of J_up_ and I_pCa_ (K_o_=7mM, f_KATP_=0.06, -25% I_Na_ & I_CaL_, -20% I_NaK_ & I_NCX_, +40% I_NaL_) (solid) and (E, G) ischaemia with impairment of J_up_ and I_pCa_ (K_o_=7mM, f_KATP_=0.06, -25% I_Na_ & I_CaL_, -20% I_NaK_ & I_NCX_, +40% I_NaL_, -30% J_up_ & I_pCa_) (solid). (B, F) Changes in Ca^2+^ transient durations measured at 90% decay (ΔCaT90) (left) and Ca^2+^ transient amplitudes (ΔCaTamp) (right) undergone for each respective condition. (D, H) Changes in AP duration measured at 90% repolarisation (APD90) (left) and maximal upstroke velocities (Δdv/dt_max_) (right) undergone for each respective condition.

1. **Supplementary References**

1. Passini E, Mincholé A, Coppini R, Cerbai E, Rodriguez B, Severi S, Bueno-Orovio A. Mechanisms of pro-arrhythmic abnormalities in ventricular repolarisation and anti-arrhythmic therapies in human hypertrophic cardiomyopathy. *J Mol Cell Cardiol* 2016;**96**:72–81.

2. Doste R, Coppini R, Bueno-Orovio A. Remodelling of potassium currents underlies arrhythmic action potential prolongation under beta-adrenergic stimulation in hypertrophic cardiomyopathy. *J Mol Cell Cardiol* 2022;**172**:120–131.

3. Coppini R, Ferrantini C, Yao L, Fan P, Lungo M Del, Stillitano F, Sartiani L, Tosi B, Suffredini S, Tesi C, Yacoub M, Olivotto I, Belardinelli L, Poggesi C, Cerbai E, Mugelli A. Late Sodium Current Inhibition Reverses Electromechanical Dysfunction in Human Hypertrophic Cardiomyopathy. *Circulation* 2013;**127**:575–584.

4. Coppini R, Ferrantini C, Aiazzi A, Mazzoni L, Sartiani L, Mugelli A, Poggesi C, Cerbai E. Isolation and Functional Characterization of Human Ventricular Cardiomyocytes from Fresh Surgical Samples. *J Vis Exp* 2014.

5. Hegyi B, Chen-Izu Y, Izu L, Banyasz T. Altered K+ current profiles underlie cardiac action potential shortening in hyperkalemia and β adrenergic stimulation. *Can J Physiol Pharmacol* 2019;**97**.

6. Moréna H, Janse MJ, Fiolet JW, Krieger WJ, Crijns H, Durrer D. Comparison of the effects of regional ischemia, hypoxia, hyperkalemia, and acidosis on intracellular and extracellular potentials and metabolism in the isolated porcine heart. *Circ Res* 1980;**46**:634–646.

7. Ferrero JM, Trenor B, Saiz J, Montilla F, Hernandez V. Electrical activity and reentry in acute regional ischemia: insights from simulations. *Conf Proc IEEE Eng Med Biol Soc*. 2003. p17–20.

8. Martinez-Navarro H, Zhou X, Bueno-Orovio A, Rodriguez B. Electrophysiological and anatomical factors determine arrhythmic risk in acute myocardial ischaemia and its modulation by sodium current availability. *Interface Focus* 2020;**11**.

9. Dutta S, Mincholé A, Quinn TA, Rodriguez B. Electrophysiological properties of computational human ventricular cell action potential models under acute ischemic conditions. *Prog Biophys Mol Biol* 2017;**129**:40–52.

10. Carpio EF, Gomez JF, Rodríguez-Matas JF, Trenor B, Ferrero JM. Analysis of vulnerability to reentry in acute myocardial ischemia using a realistic human heart model. *Comput Biol Med* 2022;**141**:105038.

11. Carpio EF, Gomez JF, Rodríguez-Matas JF, Trenor B, Ferrrero JM. Computational Analysis of Vulnerability to Reentry in Acute Myocardial Ischemia. *Comput Cardiol*. 2020. p1–4.

12. Coppini R, Mazzoni L, Ferrantini C, Gentile F, Pioner JM, Laurino A, Santini L, Bargelli V, Rotellini M, Bartolucci G, Crocini C, Sacconi L, Tesi C, Belardinelli L, Tardiff J, Mugelli A, Olivotto I, Cerbai E, Poggesi C. Ranolazine Prevents Phenotype Development in a Mouse Model of Hypertrophic Cardiomyopathy. *Circ Heart Fail* 2017;**10**:e003565.

13. Janse MJ, Cinca J, Moréna H, Fiolet JW, Kléber AG, Vries GP de, Becker AE, Durrer D. The ‘border zone’ in myocardial ischemia. An electrophysiological, metabolic, and histochemical correlation in the pig heart. *Circ Res* 1979;**44**:576–588.

14. Taggart P, Sutton PMI, Opthof T, Coronel R, Trimlett R, Pugsley W, Kallis P. Inhomogeneous Transmural Conduction During Early Ischaemia in Patients with Coronary Artery Disease. *J Mol Cell Cardiol* 2000;**32**:621–630.
